# Serum metabolomic profiles associated with subclinical and clinical cardiovascular phenotypes in people with type 2 diabetes

**DOI:** 10.1186/s12933-022-01493-w

**Published:** 2022-04-27

**Authors:** Zhe Huang, Lucija Klaric, Justina Krasauskaite, Stela McLachlan, Mark W. J. Strachan, James F. Wilson, Jackie F. Price

**Affiliations:** 1 Centre for Global Health, Usher Institute of Population Health Sciences and Informatics, University of Edinburgh, Edinburgh, UK; 2 MRC Human Genetics Unit, MRC Institute of Genetics and Cancer, University of Edinburgh, Western General Hospital, Edinburgh, UK; 3 Metabolic Unit, Western General Hospital, Edinburgh, UK

**Keywords:** Atherosclerosis, Cardiovascular diseases, Glycolysis, Lactate, Lipidomics, Metabolomics, Type 2 diabetes

## Abstract

**Background:**

Atherosclerotic cardiovascular diseases (CVD) is the leading cause of death in diabetes, but the full range of biomarkers reflecting atherosclerotic burden and CVD risk in people with diabetes is unknown. Metabolomics may help identify novel biomarkers potentially involved in development of atherosclerosis. We investigated the serum metabolomic profile of subclinical atherosclerosis, measured using ankle brachial index (ABI), in people with type 2 diabetes, compared with the profile for symptomatic CVD in the same population.

**Methods:**

The Edinburgh Type 2 Diabetes Study is a cohort of 1,066 individuals with type 2 diabetes. ABI was measured at baseline, years 4 and 10, with cardiovascular events assessed at baseline and during 10 years of follow-up. A panel of 228 metabolites was measured at baseline using nuclear magnetic resonance spectrometry, and their association with both ABI and prevalent CVD was explored using univariate regression models and least absolute shrinkage and selection operator (LASSO). Metabolites associated with baseline ABI were further explored for association with follow-up ABI and incident CVD.

**Results:**

Mean (standard deviation, SD) ABI at baseline was 0.97 (0.18, *N* = 1025), and prevalence of CVD was 35.0%. During 10-year follow-up, mean (SD) change in ABI was + 0.006 (0.178, *n* = 436), and 257 CVD events occurred. Lactate, glycerol, creatinine and glycoprotein acetyls levels were associated with baseline ABI in both univariate regression [*β*s (95% confidence interval, CI) ranged from − 0.025 (− 0.036, − 0.015) to − 0.023 (− 0.034, − 0.013), all *p* < 0.0002] and LASSO analysis. The associations remained nominally significant after adjustment for major vascular risk factors. In prospective analyses, lactate was nominally associated with ABI measured at years 4 and 10 after adjustment for baseline ABI. The four ABI-associated metabolites were all positively associated with prevalent CVD [odds ratios (ORs) ranged from 1.29 (1.13, 1.47) to 1.49 (1.29, 1.74), all *p* < 0.0002], and they were also positively associated with incident CVD [ORs (95% CI) ranged from 1.19 (1.02, 1.39) to 1.35 (1.17, 1.56), all *p* < 0.05].

**Conclusions:**

Serum metabolites relating to glycolysis, fluid balance and inflammation were independently associated with both a marker of subclinical atherosclerosis and with symptomatic CVD in people with type 2 diabetes. Additional investigation is warranted to determine their roles as possible etiological and/or predictive biomarkers for atherosclerotic CVD.

**Supplementary information:**

The online version contains supplementary material available at 10.1186/s12933-022-01493-w.

## Background

Atherosclerosis and its major clinical manifestation-cardiovascular diseases (CVD) is the leading cause of death globally. Individuals with type 2 diabetes are several times more likely to develop CVD and suffer heavier health and economic burden than the general population [1, 2]. Although increased cardiovascular risk is likely attributable, at least in part, to dyslipidaemia, inflammation and oxidative stress [3], the association of CVD with detailed metabolic status at the molecular level in type 2 diabetes populations remains unclear.

As a recently emerging field of systematic study, metabolomics is proximal to disease phenotypes and offers opportunities to depict a high-definition snapshot of numerous small low-molecular-weight metabolites in a single biological sample, therefore facilitating elucidation of complex disease pathobiology and identification of predictive biomarkers [4, 5]. The metabolomic profile of CVD has been described in several studies, but these have been mainly based on general populations. Although results were highly heterogeneous, especially given the wide variety of metabolomic platforms used, some subclasses of lipids, amino acids and dicarboxylacylcarnitines have shown strong association with clinical CVD and have provided additional predictive value to development of CVD over traditional cardiovascular risk factors [6, 7]. Whilst these findings in the general population show the potential value of metabolomics studies for vascular research, only a limited number of similar studies have explored metabolomic characteristics of CVD in people with type 2 diabetes, and existing studies were primarily small-scale case-control studies [8, 9].

Critically, the atherosclerotic process develops silently for several decades, providing a window for early intervention to prevent clinical CVD. Such asymptomatic disease is particularly prevalent in people with type 2 diabetes [10]. Several markers have been developed to assist in the detection of early asymptomatic CVD. Metabolomic changes associated with these markers are important to identify because of their potential to aid understanding of the early development of CVD and also their potential use in early risk prediction. Ankle-branchial index (ABI, ratio of systolic blood pressure in the ankle to that in the arm) has particularly proved to be a good measure of systemic atherosclerosis and serves as a risk indicator even in patients with CVD [11]. However, few studies have explored the metabolomic profile of ABI in either general populations or people with diabetes, and in those metabolomic studies where atherosclerosis was evaluated by other markers (e.g., pulse wave velocity), populations with diabetes were mainly small Asian groups [12–14].

The aim of the current research was to explore the metabolomic profile for atherosclerotic CVD in people with type 2 diabetes, focusing on both ABI as a measure of subclinical disease and coronary/cerebrovascular disease as a measure of clinical CVD. We aimed to identify novel individual metabolites worthy of further exploration as potential causal and/or predictive cardiovascular biomarkers in people with type 2 diabetes.

## Methods

### Study population

The Edinburgh Type 2 Diabetes Study (ET2DS) is a prospective cohort study of 1066 older men and women with confirmed type 2 diabetes at baseline (2006/2007) living in Lothian, Scotland. Details of recruitment have been described elsewhere [15]. Briefly, individuals aged 60–75 years were recruited to form a representative sample from the Lothian Diabetes Register where almost all individuals with type 2 diabetes in Lothian are recorded. Questionnaires and physical examination were used to collect data at baseline, and again at years 4 (*n* = 831) and 10 (*n* = 581) when participants were invited to follow up physical investigation [16]. Data on cardiovascular events was obtained on all participants through linkage with the Information Services Division of National Health Service Scotland at baseline and years 4 and 8, and hospital discharge and deaths records were further updated using hospitalization records and scrutiny of death records at National Records of Scotland at year 10 (*n* = 1066).

Given ABI > 1.3 is widely regarded as a sign of the presence of medial arterial calcification which has different clinical characteristics from atherosclerosis, individuals with ABI > 1.3 at any time point were excluded. This study finally included 1,025 individuals for principal analyses who do not have missing data on metabolomics and/or baseline ABI measurement. For longitudinal analyses on follow-up ABI, only individuals with available baseline and corresponding follow-up ABI measurement were included (*n* = 731 and 436 for ABI at years 4 and 10 respectively). Ethical permission was granted by the Lothian Medical Research Ethics Committee and written informed consent was obtained from all participants.

### Metabolomic profile of serum sample

Baseline fasting venous blood samples were collected to measure serum metabolomics data using a high-throughput nuclear magnetic resonance (NMR) platform (Nightingale, Helsinki, Finland) which has been described in detail by Soininen etc. and has been used in a number of large-scale epidemiological studies [17, 18]. Overall, 228 serum metabolites were reported in the form of either absolute concentrations or ratios. These mainly included lipid particles and subclasses (corresponding size, density, components), fatty acids, glycolysis related metabolites, amino acids, ketone bodies, fluid balance molecules and inflammation marker. Some lipid ratios were reported as infinite where the concentration of the denominator (or part of the denominator) was missing due to being below the minimum level of detection. In this instance, we replaced the missing values with a value equal to half of the minimum concentration recorded in the dataset for the affected metabolite, and recalculated the ratio using this estimated value.

### ABI and CVD events

To measure ABI, participants were asked to rest for at least 15 min and an aneroid sphygmomanometer and a doppler probe (Dopplex® advanced pocket Doppler, Huntleigh Healthcare Ltd., Cardiff, UK) were used to measure both right and left brachial, posterior tibial and dorsalis pedis systolic blood pressures (SBP) in the supine position [15]. ABI was calculated by dividing the lowest ankle SBP by the highest brachial SBP. ABI was measured at baseline, years 4 and 10 by specially trained research staff using identical standard operating procedures.

Detailed criteria for prevalent and incident cardiovascular events in ET2DS have been described elsewhere [19]. Briefly, for the purposes of this analysis, CVD refers to myocardial infarction (MI), angina, coronary intervention, stroke and transient ischemic attack. Prevalent cardiovascular events were defined according to baseline data from self-completion questionnaires, ECGs and historical hospital discharge records, and incident events were ascertained by criteria based on collection of data at research clinics at years 4 and 10 together with comprehensive scrutiny of clinical and hospital discharge records and death certificates during the follow-up.

### Statistical analysis

Characteristics of the study participants were presented as means and standard deviations (SDs) or medians and interquartile ranges (IQRs) for continuous variables and percentages for categorical variables. Normal distribution of metabolites and residuals of regression models was visually checked, and raw levels of metabolites were standardized by subtracting the mean and then dividing by the SD for each metabolite. In univariate analysis where baseline ABI was the outcome, each metabolite was modelled using linear regression adjusting for age and sex (Model 1). The significance threshold was set to 0.0002 (0.05/228, Bonferroni corrected for 228 metabolites) to account for multiple comparisons. Sensitivity analysis where existing CVD cases were excluded was performed to explore the stability of the key metabolites in the metabolomic profile of baseline ABI. Metabolites associated with baseline ABI were further explored in terms of association with follow-up ABI, and follow-up ABI was analysed as the outcome using 3 different approaches: (1) absolute changes in ABI during 4 and 10 years, (2) levels of follow-up ABI with adjustment for baseline ABI, and (3) slopes of interpolated lines of three measurements of ABI during 10 years for each available individual.

Additionally, given the property of high dimension and multi-collinearity of metabolomics data, least absolute shrinkage and selection operator (LASSO) was used to describe the metabolomic profile of baseline ABI and to assess the stability of results from univariate analyses. LASSO shrinks coefficients of some variables to exactly zero by adding a penalty and thus selects the most informative panel of variables, making it particularly suitable for high-dimensional metabolomics data [20]. Using *cv.glmnet* function on *glmnet* package (version 4.0–2) on R, we applied a five-fold cross-validation incorporating 228 metabolites to obtain the optimum tuning parameter *λ* which gave the minimum mean error/deviance in the linear/logistic regression model. Then, the reduced panel of metabolites together with their coefficients were reported to assess the consistency with metabolites identified in univariate analyses.

An age- and sex-adjusted logistic regression model was performed to select significant metabolites associated with prevalent CVD in univariate analysis, following with LASSO analysis to uncover metabolomic profiles of prevalent CVD. To further explore whether the association between identified metabolites and baseline ABI and/or prevalent CVD was independent of traditional CVD risk factors, we further adjusted for smoking, SBP, high-density lipoprotein (HDL)-cholesterol, total cholesterol, body mass index (BMI) and glycated haemoglobin (HbA1c) (Model 2). Moreover, the associations between the baseline ABI-associated metabolites and incident CVD were further assessed using univariate logistic regression models, and prevalent CVD was adjusted to assess the stability of observed association.

To explore if the observed association could lie on the pathophysiological pathways of traditional CVD risk factors, inflammation markers and drugs use, the relationships between key metabolites and important covariates were assessed using Pearson correlations. Finally, odds ratios (ORs) and 95% confidence interval (CI) of 228 metabolites were extracted from related univariate analyses with age and sex adjustment where low ABI (< 0.9, a cut-point used previously for identifying individuals with poor cardiovascular prognosis) and CVD at baseline served as the outcome separately. Spearman correlation coefficient was calculated to quantify the concordance between the metabolomic profile of low ABI and CVD at baseline. All analyses were performed using R version 4.0.3 (R Foundation for Statistical Computing, Vienna, Austria).

## Results

### Characteristics of the study population

Major demographic and clinical characteristics of this study population at baseline, together with a description of the main outcome phenotypes, are shown in Table 1 (see Additional file 1: Figure S1 for further details on change in ABI). Mean ABI (SD) at baseline was 0.97 (0.18), and mean (SD) changes of ABI among available individuals over 4 and 10 years were + 0.001 (0.152) and + 0.006 (0.178) respectively. Prevalence of CVD was 35.0%, with 257 cardiovascular events (123 new and 134 recurrent events) developing during 10 years of follow-up (detailed frequency of CVD constituent endpoints are shown in Additional file 1: Table S1). Mean values of serum metabolomics at baseline are shown in  Additional file 1: Table S2. There was considerable inter-correlation between individual metabolites, particularly among lipid-related metabolites, with correlations ranging from − 0.998 to 1.000 (see Additional file 1: Figure S2).

**Table 1:** Demographic and Clinical characteristics of the study population (N = 1025)

Characteristics	Available individuals
At Baseline	
Age (years)	67.8 (4.2)
Male	520 (50.7)
Smoking	–
Non–smoker	398 (38.8)
Ever–smoker	486 (47.4)
Current smoker	141 (13.8)
SBP (mmHg)	133.2 (16.4)
BMI (kg/m^2^)	31.4 (5.6)
HDL–cholesterol (mmol/l)	1.3 (0.4)
Total Cholesterol (mmol/l)	4.3 (0.9)
eGFR (mL/min/1.73 m^2^) [Median (IQR)]	60.0 (14.0)
Glucose (mmol/l)	7.6 (2.1)
HbA1c (%)	7.4 (1.1)
HbA1c (mmol/mol)	57.5 (12.4)
Duration of diabetes (years) [Median (IQR)]	6.0 (8.0)
Lipid lowering drug use	881 (86.1)
ABI	0.97 (0.18)
Prevalent CVD	359 (35.0)
Follow–up	
ABI at Year 4 (all available individuals)	0.97 (0.18)
ABI at Year 4 (individuals with ABI measured at both Year 4 and Year 10)	0.99 (0.16)
ABI at Year 10	1.00 (0.18)
CVD events over 10 years	257 (25.1)

Data are mean (SD) for continuous variables and *n* (%) for categorical variables unless stated otherwise. Missing cases for variables of interest were as follows: SBP = 2 cases, BMI = 1 case, HDL-cholesterol = 6 cases, Total cholesterol = 6 cases, HbA1c (%) = 9 cases

### Metabolomic profiles associated with ABI

**Fig. 1:**
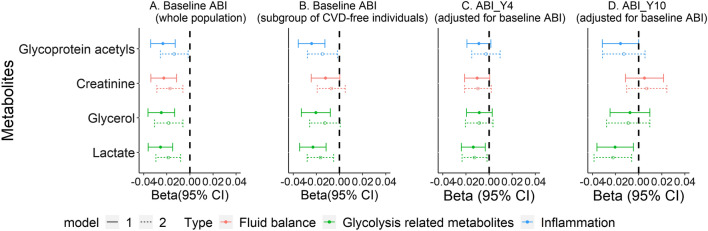
Association between the four key metabolites and baseline and follow-up ABI. **A** Baseline ABI (whole population, *N* = 1025), **B** Baseline ABI (subgroup of CVD-free individuals at baseline, *n* = 666), **C** ABI at Year 4 (adjusted for baseline ABI, *n* = 731), **D** ABI at Year 10 (adjusted for baseline ABI, *n* = 436). The solid lines represent Model 1 (adjusted for age and gender), and the dotted lines represent Model 2 (model 1 plus SBP, smoking, HDL-cholesterol, total cholesterol, BMI and HbA1c)

Of all 228 metabolites tested for association with baseline ABI (see Additional file 1: Table S3 for detailed *p* values and *β*s), four were significant (*p* < 0.0002) in univariate analysis adjusted for age and gender. These were lactate, glycerol, creatinine and glycoprotein acetyl [*β*s (95%CI): − 0.025 (− 0.036, − 0.015), − 0.025 (− 0.036, − 0.013), − 0.023 (− 0.034, − 0.012), − 0.023 (− 0.034, − 0.013), respectively, see model 1 in Fig. 1A and Additional file 1: Table S4]. The same four metabolites were also associated with ABI in LASSO analysis (see Additional file 1: Table S5), in addition to another three metabolites (i.e., histidine, total lipids and total cholesterol in small HDL). Unsurprisingly, effect sizes for the association of these metabolites with ABI were attenuated after adjusting for traditional CVD risk factors, but all associations remained in the same direction and nominally significant (model 2 in Fig. 1A). We further exploratorily adjusted for estimated glomerular filtration rate (eGFR), lipid-lowering drug use and duration of diabetes in model 3a, lactate remained significantly associated with baseline ABI with *p* < 0.0002 (Additional file 1: Table S4).

A total of 666 individuals were included in sensitivity analysis on participants without clinical CVD at baseline. While the association between creatinine and ABI in the sub-group was not statistically significant, lactate and glycoprotein acetyl were significantly associated with ABI with *p* < 0.0002 (*β*s: − 0.023 and − 0.024, respectively, Fig. 1B). After further adjustment for traditional CVD risk factors, lactate and glycoprotein acetyls remained nominally associated with baseline ABI.

In analyses focusing on the association between the four metabolites identified above with change in ABI during follow-up, lactate showed nominally significant association with ABI measured at year 4 (*β*: − 0.014, see Fig. 1C and Additional file 1: Table S6) and year 10 (*β*: − 0.020, see Fig. 1D and Additional File 1: Table S6) following adjustment for baseline ABI, with associations attenuated after adjusting for other covariates. Borderline statistically significant associations were observed in similar analyses for follow-up ABI and the other three metabolites (e.g., for creatinine, *p* = 0.06). No significant associations were found in analyses where change in ABI was modelled as the absolute difference between follow-up ABI and baseline ABI, or the slope of interpolated line of three measurements of ABI (Additional file 1: Figure S3).

### Metabolomic profiles associated with clinical CVD

**Fig. 2:**
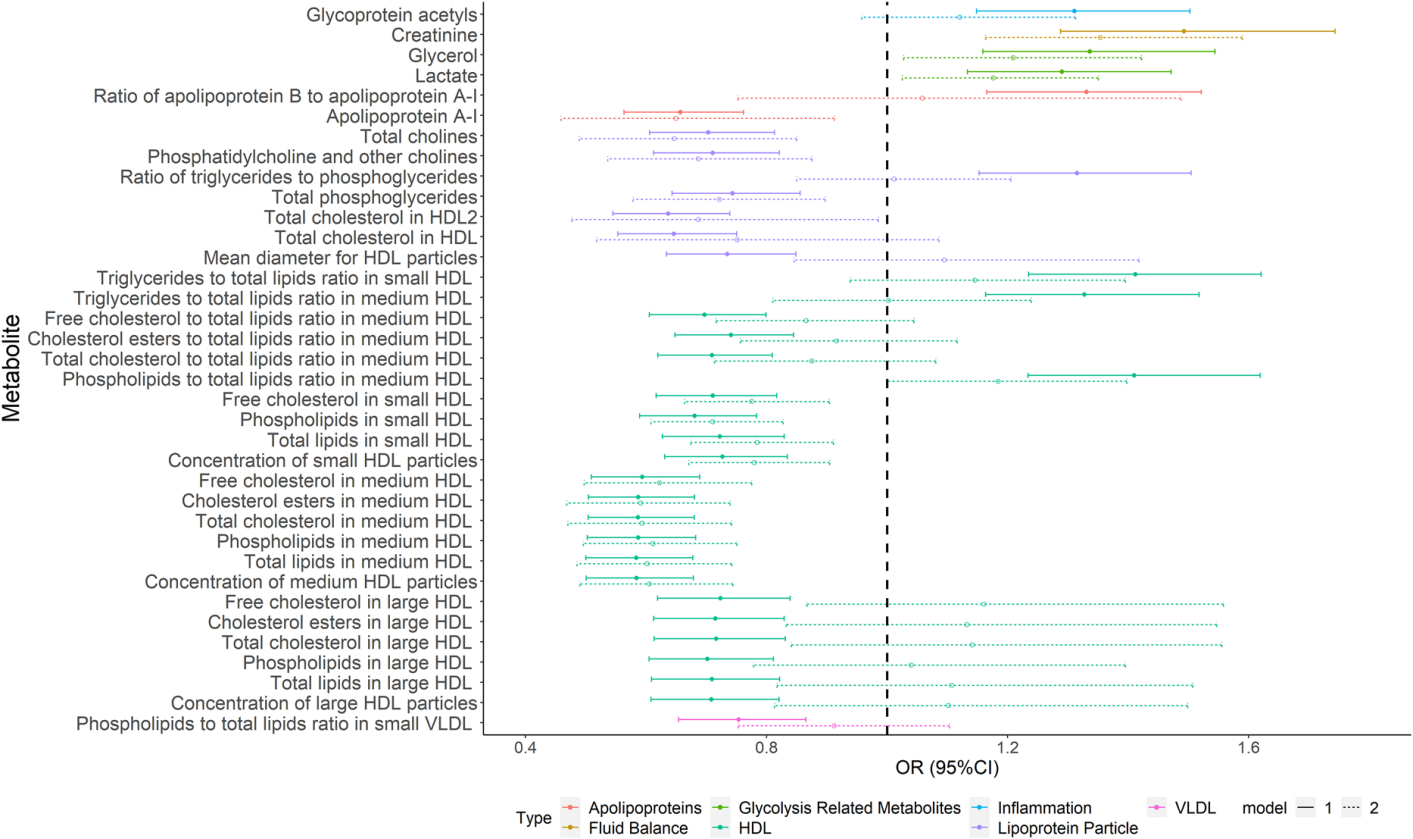
Association between key metabolites and prevalent CVD. The solid lines represent Model 1 (adjusted for age and gender) and the dotted lines represent Model 2 (model 1 plus SBP, smoking, HDL-cholesterol, total cholesterol, BMI and HbA1c)

In univariate analysis, 36 metabolites were associated with prevalent CVD (*p* < 0.0002), primarily metabolites of HDL subclasses and lipoprotein particles (see Fig. 2 and Additional file 1: Table S7). Six of these were also identified in LASSO analysis (see Additional file 1: Table S8), in addition to seventeen exclusive metabolites (mainly components of HDL and amino acids). Associations between prevalent CVD and some lipids subclasses were substantially weakened following adjustment for CVD risk factors, but 19 metabolites remained nominally significantly associated. Particularly, creatinine and components of medium HDL lipids were strongly associated with CVD (Fig. 2). All four metabolites associated with baseline ABI were positively associated with prevalent CVD and showed good consistency in terms of direction of association [ORs (95%CI): 1.29 (1.13, 1.47), 1.34 (1.16, 1.54), 1.49 (1.29, 1.74) and 1.31 (1.15, 1.50), respectively, all *p* < 0.0002]. A one SD increase of serum creatinine showed the strongest association with prevalent CVD, with OR of 1.34 (95% *CI*:1.16–1.54), even following adjustment for traditional CVD risk factors, but the association became nonsignificant after we further adjusted for eGFR in model 3a (Additional file 1: Table S7).

**Fig. 3:**
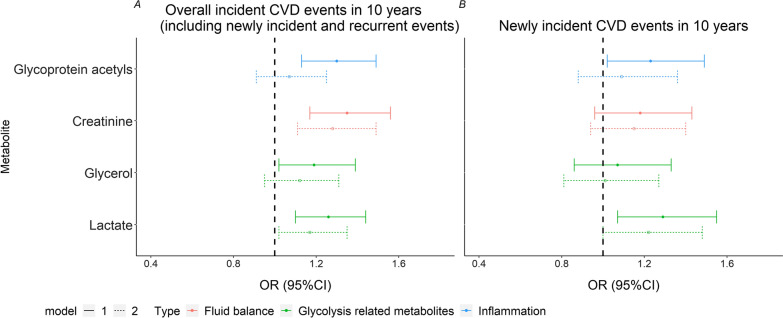
Association between the four key metabolites and incident CVD events in 10 years. **A** Overall incident CVD events (including newly incident and recurrent CVD) during 10-year follow-up period (*N* = 1025). **B** Newly incident CVD events during 10-year follow-up period (*n* = 666). The solid lines represent Model 1 (adjusted for age and gender) and the dotted lines represent Model 2 (model 1 plus SBP, smoking, HDL-chlesterol, total cholesterol, BMI and HbA1c)

The four metabolites also showed nominally positive associations with the occurrence of cardiovascular events during 10-year follow-up [ORs (95%CI): 1.26 (1.10, 1.44), 1.19 (1.02, 1.39), 1.35 (1.17, 1.56) and 1.30 (1.13, 1.49) respectively], although unsurprisingly, associations were substantially attenuated when adjusted for CVD risk factors (Fig. 3A and Additional file 1: Table S9). After existing CVD cases at baseline were excluded (*n* = 666), lactate and glycoprotein acetyls showed some association with newly incident CVD (ORs = 1.29 and 1.23, and *p* = 0.007 and 0.027, respectively), though the associations were not significant when further adjusted for other covariates (Fig. 3B and Additional file 1: Table S9). We also adjusted for prevalent CVD to evaluate the stability of observed associations, and most of associations were still nominal significant despite being attenuated (Additional file 1: Table S10).

### Correlation between important covariates and key metabolites associated with baseline ABI and/or prevalent CVD

We next calculated Pearson correlation coefficients between important covariates and key metabolites (Additional file 1: Figure S4). As expected, metabolites of HDL subclasses, lipid particles, and apolipoproteins showed moderate to strong correlations with classical lipids (e.g., HDL-cholesterol and total cholesterol). Creatinine showed strong inverse correlation with eGFR, and glycerol was moderately correlated with BMI. Of note, lactate showed weak correlations with all covariates.

### Concordance between metabolomic profile of low ABI and CVD at baseline

**Fig. 4:**
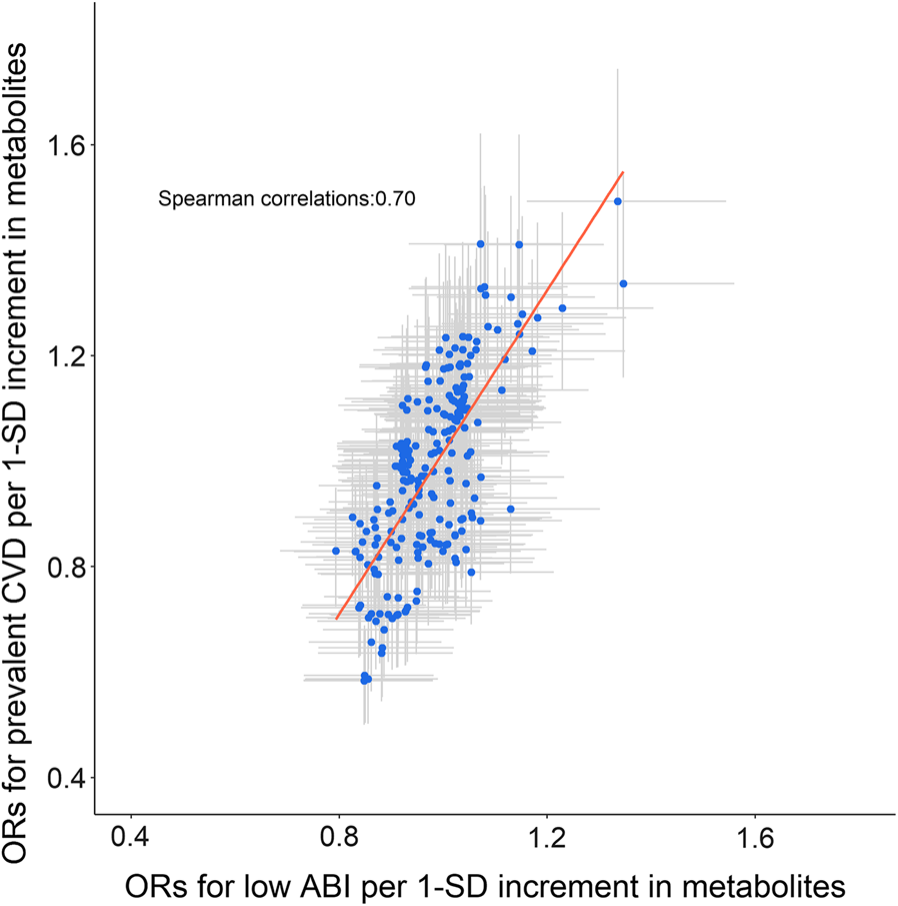
Concordance of metabolomic profile of low ABI and prevalent CVD at baseline. Each spot represents a metabolite, and values in x-axis represents ORs associated with low ABI (i.e., ABI < 0.9) and values in y-axis represents ORs associated with prevalent CVD in model 1. Grey bars were 95% CIs of related ORs in x-axis and y-axis, and the red solid line simulated the linear pattern of these spots

Figure 4 shows the extent to which there was concordance between the metabolomic profiles for low ABI (< 0.9) and CVD at baseline. The Spearman correlation coefficient was 0.70, indicating a generally good concordance.

## Discussion

In this study, we described and compared the association of serum NMR metabolites with ABI, change in ABI and with both prevalent and incident symptomatic CVD in men and women with type 2 diabetes. Four metabolites (lactate, glycerol, creatinine and glycoprotein acetyls) were associated with baseline ABI, and they were also associated with prevalent CVD. Lactate particularly emerged as a potentially promising candidate biomarker of atherosclerotic burden in type 2 diabetes due to an association with ABI and CVD in both cross-sectional and longitudinal analyses. Interestingly, associations of glycerol, creatinine, and glycoprotein acetyls with baseline ABI were also evident after excluding existing CVD cases or adjustment for CVD risk factors, although statistical significance became borderline, potentially partly because of the smaller sample size.

Besides these main findings, we found that several metabolites belonging to medium and small HDL subclasses were associated with prevalent CVD, independently of traditional CVD risk factors. Attenuation in regression coefficients following adjustment for traditional CVD risk factors suggests such metabolites could lie on common pathophysiological pathways underlying the development of atherosclerotic disease, and strong correlation between the metabolomic profile of low ABI and CVD at baseline points to both shared and unique risk factors for these two related conditions. Whilst our findings suggest that the metabolomic characteristics of atherosclerosis in people with type 2 diabetes might alter as the disease progresses, the precise role (if any) that these metabolites play in the development of CVD needs further investigation, for which multi-omics studies and external populations are likely to be helpful.

Of all metabolites studied, lactate showed consistent, independent associations across all phenotypes and had weak correlations with CVD risk factors, highlighting specific potential as an independent atherosclerotic biomarker in diabetes. As an end product of anaerobic glycolysis, lactate is a good indicator of mitochondrial dysfunction which is a major characteristic of type 2 diabetes [21, 22]. Mitochondrial dysfunction would promote the production of reactive oxygen species which would exacerbate atherosclerosis, and lead to apoptosis thereby accelerating plaque rupture and increasing risk of ischemic CVD [21]. Given this, the association could be partly attributed to the severity of insulin resistance reflected by lactate. However, lactate’s roles in other pathways (e.g., regulating metabolism) should also be noted because the association remained significant after adjusting for HbA1c [23]. Of note, almost 60% of individuals in the ET2DS were using metformin at baseline, and lactic acidosis is a recognised complication of metformin therapy [24] although metformin is thought to have a protective role on cardiovascular outcomes [25]. To assess the effect of metformin use on the association between lactate and baseline ABI, metformin use was adjusted in model 3b (data was not shown), and there was only a slight alteration in the association strength following such adjustment (*β* changed from − 0.0187 to − 0.0192).

In a previous study with a similar mean age to ET2DS but with only a small proportion of individuals with diabetes (14.3%), lactate was associated with two subclinical markers of atherosclerosis and incident CVD [26]. However, the association lost statistical significance after adjusting for CVD risk factors, including diabetes, and it is possible that lactate might function differently in people with and without diabetes in relation to indicating atherosclerotic risk. This would be consistent with marginally significant associations between markers of atherosclerosis and lactate in another two studies where only a few individuals had diabetes [27, 28].

Glycoprotein acetyl is a NMR composite biomarker of systemic inflammation, reflecting abundance of mobile N-acetyl sugar groups on glycoproteins in blood [29], which has been reported to show strong positive association with carotid/peripheral atherosclerosis [30, 31]. We also found glycoprotein acetyl to be negatively associated with ABI, but the association was reduced by adjustment for CVD risk factors, suggesting possible contribution to common pathways involving traditional lipids and inflammation. Analogously, previous studies based in general population revealed the predictive value of glycoprotein acetyls for CVD [32], and the positive association was also replicated in ET2DS, even though it was substantially diminished by adjustment for routinely measured lipids. It has been suggested glycoprotein acetyls might be a good indicator for long-term prognosis for diabetes with peripheral artery disease [33].

Creatinine is an established marker of kidney function, and chronic kidney disease is a well-known risk factor for CVD [34]. Interestingly, we found the association of creatinine with both ABI and CVD was more sensitive to excluding existing CVD cases than to adjusting for traditional CVD risk factors, and it is possible that vascular-related kidney damage may exacerbate the association between creatinine and CVD among CVD cases. Notably, we found creatinine was associated with incident CVD but not with change in ABI. Although this precise result has not been reported in other studies, a study in individuals with diabetes reported an association of albuminuria with early but not late carotid atherosclerotic lesions [35], raising the possibility of different markers of kidney dysfunction reflecting risk of different stages of atherosclerosis. Previous study also reported a positive association of creatinine with MI but not with stroke [32], and Juonala etc. [28] found creatinine was inversely associated with PWV but not with carotid intima media thickness. It may be that the association of creatinine with atherosclerosis or CVD may be specific to the precise markers or subtypes of disease studied.

Low-density lipoprotein (LDL)-cholesterol and HDL-cholesterol are well-established risk factors for CVD, but little is known about how particle concentrations and lipids components are associated with atherosclerotic phenotypes in diabetes. In our study, medium and large HDL-cholesterol showed inverse association with CVD, but no statistically significant results were found for subclasses of LDL. The absence of association between smaller HDL-cholesterol and CVD was also reported in general populations [32, 36], but these studies found cholesterol components of LDL and triglyceride components of all lipids were risk factors for CVD. As MI and stroke have different, even opposing, metabolomic profiles [32, 37], some association might be masked in ET2DS where CVD was considered as an overall phenotype.

Previous metabolomic studies of atherosclerosis in people with diabetes, which are limited to small-scale studies [12, 13, 38], include a recent study among 209 people with type 2 diabetes in Japan. It found indoxyl sulfate was significantly associated with brachial-ankle pulse wave velocity (baPWV) in both exploratory and validation datasets, but the association of mannitol, mesoerythritol and pyroglutamic acid with baPWV failed to be replicated in the validation dataset [12]. Notably, the same researchers also reported significant associations of indoxyl sulfate with another two markers of subclinical atherosclerosis in Japanese people with diabetes [38]. Nevertheless, similar studies from other ethnics are relatively scarce. Chevli etc. [39] found fatty acid, androgenic steroids and other sub-pathways were associated with subclinical atherosclerosis in a family-based diabetes-enriched population and the associations vary in African and European Americans. Moreover, in studies on the general population, various chemical compounds of lysophosphatidylcholine have been frequently reported to be associated with atherosclerosis [40, 41]. However, we were unable to test these metabolites in ET2DS given the different metabolomics platforms used.

A strength of our study is the well-characterized and representative ET2DS cohort with both baseline and follow-up data, which enabled us to explore the metabolomic profile of changes in ABI and cross-sectional associations. Additionally, given collinearity of metabolomics data, modern statistical techniques were applied in this study to further confirm the stability of identified metabolites in univariate analyses. Whilst our study is limited by a relatively small sample size (especially in longitudinal analyses for ABI at Year 10 where only a half of population was alive and visited the clinic), which restricted the statistical power, it remains one of the largest metabolomics studies for atherosclerosis among people with type 2 diabetes. To minimize the effect of potential confounding, we adjusted for a variety of CVD risk factors, but it is important to note the potential for residual confounding by unmeasured co-variates, such as diet. Furthermore, prevalent CVD at baseline restricts our ability to claim identified metabolites for ABI could serve as early biomarkers for subsequent CVD, whereas some key metabolites (such as lactate) remained significantly associated in the subgroup analysis with CVD-free individuals despite a reduced sample size. Critically, validation of our findings in an independent dataset is lacking, so replication will be required in external populations, to both confirm or refute our findings and to assess their generalizability.

In conclusion, in this study of 1,025 participants from a representative cohort of individuals with type 2 diabetes, the metabolomic profile of ABI and that of CVD were similar but not identical. Among the metabolites identified, lactate proved most promising for indicating CVD risk due to its consistent association with both ABI and CVD. Although association of some metabolites of HDL subclasses, creatinine, and glycolysis related metabolites with CVD were attenuated by adjusting for CVD risk factors, their predictive value for the development or progression of atherosclerotic cardiovascular disease is worthy of further exploration.

## Supplementary Information

**Additional file 1: Figure S1**. Distribution of changes in ABI during follow-up period. **Figure S2**. Correlation matrix of metabolites. **Figure S3**. Association between the four key metabolites and different forms of changes in ABI. **Figure S4**. Correlation matrix between covariates and key metabolites associated with baseline ABI and/or prevalent CVD. **Table S1**. Frequency of constituent endpoints for both prevalent CVD and incident CVD in ET2DS. **Table S2**. Distribution of serum metabolites of the ET2DS at baseline. **Table S3**. Association between each metabolite and baseline ABI, adjusted for age and sex. **Table S4**. Association between key metabolites and baseline ABI in univariate analysis. **Table S5**. Association between key metabolites and baseline ABI as estimated by LASSO. **Table S6**. Association between the four key metabolites and follow-up ABI in univariate analysis. **Table S7**. Association between key metabolites and prevalent CVD at baseline in univariate analysis. **Table S8**. Association between key metabolites and prevalent CVD at baseline as estimated by LASSO. **Table S9**. Association between the four key metabolites and incident CVD over 10 years in univariate analysis. **Table S10**. Association between the four baseline ABI-associated metabolites and overall incident CVD with adjustment for age, gender and prevalent CVD.

## Data Availability

The dataset analysed during current study is not publicly available due to it containing information that could compromise research participant privacy/consent, but aggregate data and analytical plan might be available from the corresponding author on reasonable request.

## Abbreviations

ABI: Ankle brachial index
baPWV: Brachial-ankle pulse wave velocity
BMI: Body mass index
CI: Confidence interval
CVD: Cardiovascular disease
eGFR: Estimated glomerular filtration rate
ET2DS: Edinburgh type 2 diabetes study
HbA1c: Glycated haemoglobin
HDL: High-density lipoprotein
IQR: Interquartile range
LASSO: Least absolute shrinkage and selection operator
LDL: Low-density lipoprotein
NMR: Nuclear magnetic resonance
MI: Myocardial infarction
OR: Odds ratio
SBP: Systolic blood pressures
SD: Standard deviation
