# Supplementary material for: Serum metabolomic profiles associated with subclinical and clinical cardiovascular phenotypes in people with type 2 diabetes
Source: Cardiovasc Diabetol. 2022 Apr 27;21:62. doi: 10.1186/s12933-022-01493-w (PMC9047374; doi:10.1186/s12933-022-01493-w)
Supplement: Supplementary file 1 — Additional file 1: Figure S1. Distribution of changes in ABI during follow-up period. Figure S2. Correlation matrix of metabolites. Figure S3. Association between the four key metabolites and different forms of changes in ABI. Figure S4. Correlation matrix between covariates and key metabolites associated with baseline ABI and/or prevalent CVD. Table S1. Frequency of constituent endpoints for both prevalent CVD and incident CVD in ET2DS. Table S2. Distribution of serum metabolites of the ET2DS at baseline. Table S3. Association between each metabolite and baseline ABI, adjusted for age and sex. Table S4. Association between key metabolites and baseline ABI in univariate analysis. Table S5. Association between key metabolites and baseline ABI as estimated by LASSO. Table S6. Association between the four key metabolites and follow-up ABI in univariate analysis. Table S7. Association between key metabolites and prevalent CVD at baseline in univariate analysis. Table S8. Association between key metabolites and prevalent CVD at baseline as estimated by LASSO. Table S9. Association between the four key metabolites and incident CVD over 10 years in univariate analysis. Table S10. Association between the four baseline ABI-associated metabolites and overall incident CVD with adjustment for age, gender and prevalent CVD. [file 12933_2022_1493_MOESM1_ESM.docx]

**Additional File**

# List of contents:

Figure S1. Distribution of changes in ABI during follow-up period.

Figure S2. Correlation matrix of metabolites.

Figure S3. Association between the four key metabolites and different forms of changes in ABI.

Figure S4. Correlation matrix between covariates and key metabolites associated with baseline ABI and/or prevalent CVD.

Table S1. Frequency of constituent endpoints for both prevalent CVD and incident CVD in ET2DS.

Table S2. Distribution of serum metabolites of the ET2DS at baseline.Table S3. Association between each metabolite and baseline ABI, adjusted for age and sex.

Table S4. Association between key metabolites and baseline ABI in univariate analysis.

Table S5. Association between key metabolites and baseline ABI as estimated by LASSO.

Table S6. Association between the four key metabolites and follow-up ABI in univariate analysis.

Table S7. Association between key metabolites and prevalent CVD at baseline in univariate analysis.

Table S8. Association between key metabolites and prevalent CVD at baseline as estimated by LASSO.

Table S9. Association between the four key metabolites and incident CVD over 10 years in univariate analysis.

Table S10. Association between the four baseline ABI-associated metabolites and overall incident CVD with adjustment for age, gender and prevalent CVD.


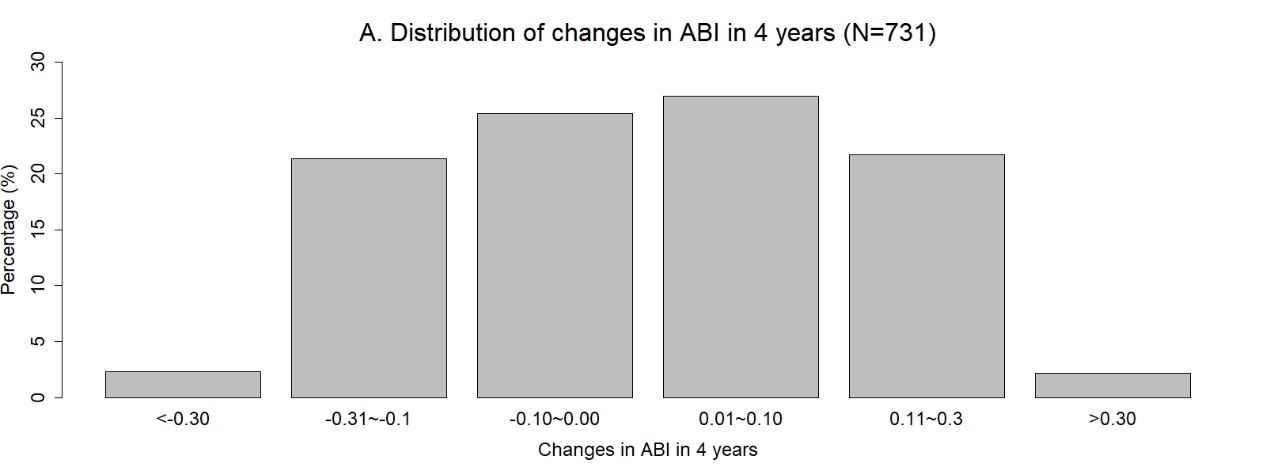


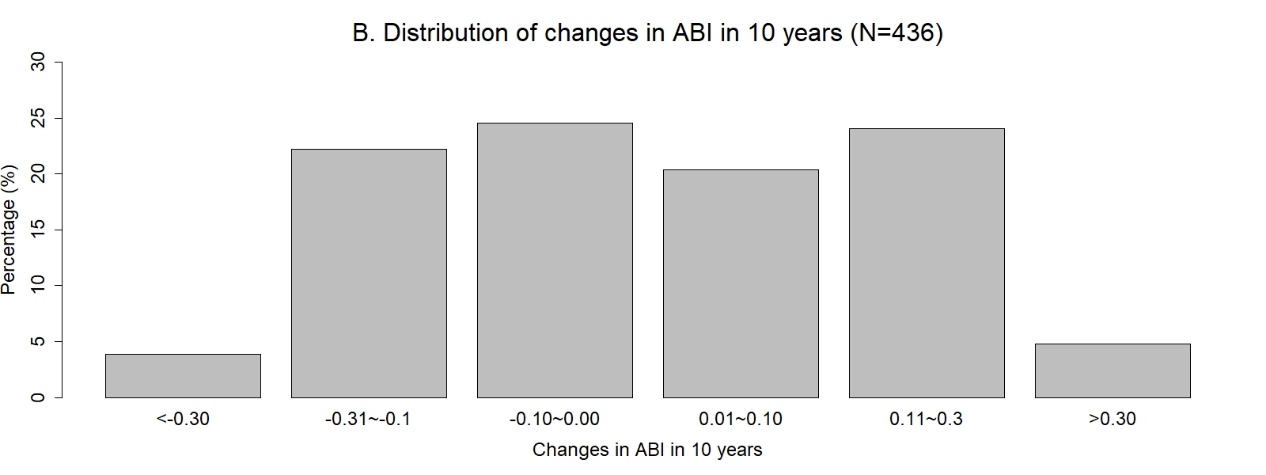


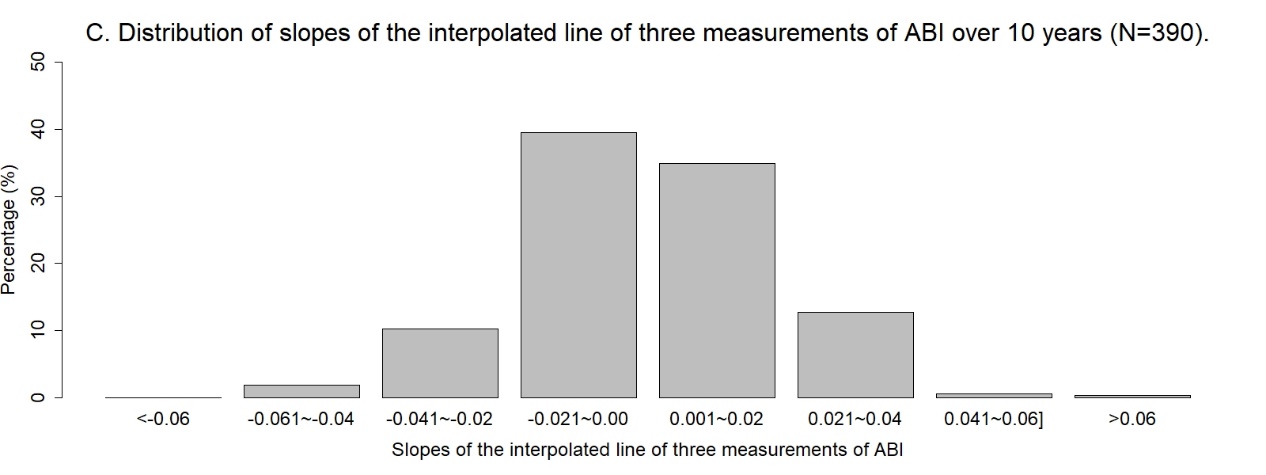


# Figure S1. Distribution of changes in ABI during follow-up period.

A. Distribution of changes in ABI in 4 years (*N*=731); B. Distribution of changes in ABI in 10 years (*N*=436); C. Distribution of slopes of the interpolated line of three measurements of ABI over 10 years (*N*=390)**.**


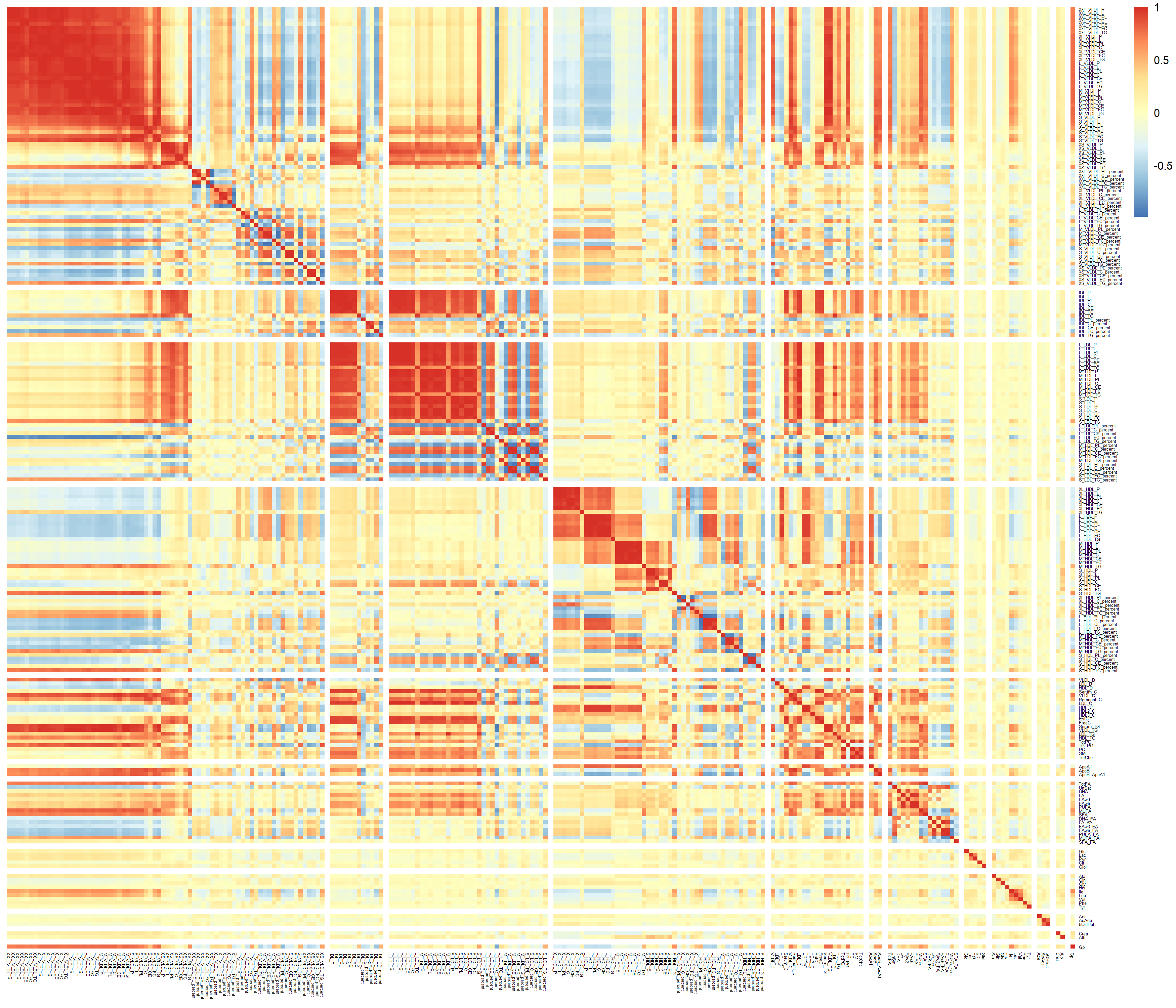


# Figure S2. Correlation matrix of metabolites.

Red colour represents positive correlation, and blue colour represents negative correlation. The darker the colour is, the stronger the correlation is.


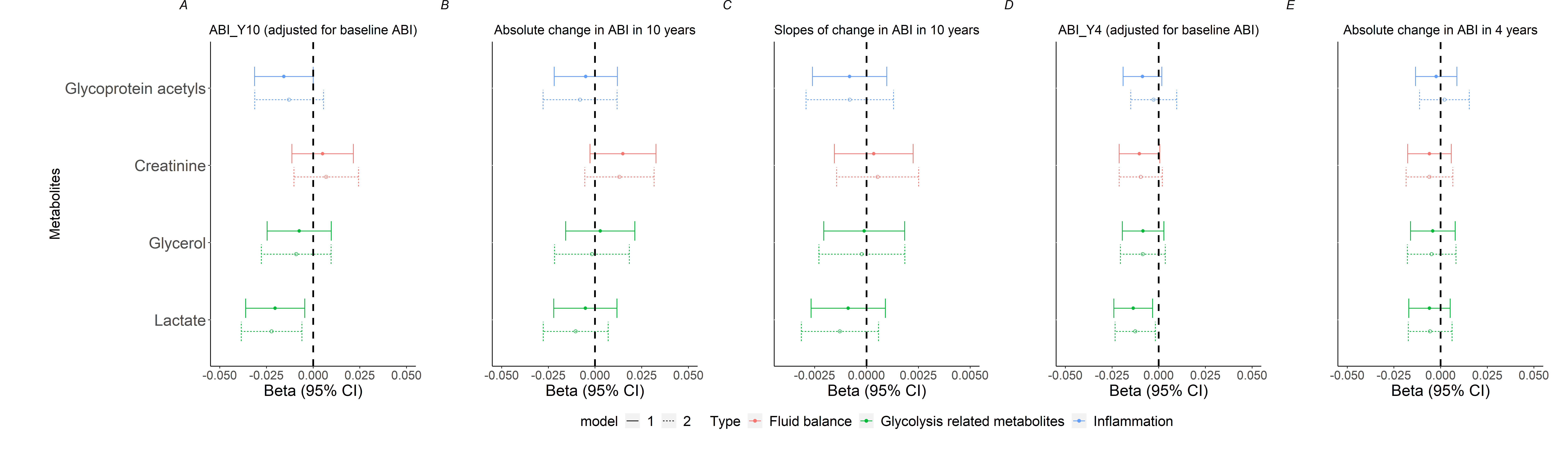


# Figure S3. Association between the four key metabolites and different forms of changes in ABI.

A. Follow-up ABI at year 10 with adjustment for baseline ABI (*N*=436). B. Absolute difference of ABI in 10 years (*N*=436). C. Slopes of three measurements of ABI in 10 years (*N*=390). D. Follow-up ABI at year 4 with adjustment for baseline ABI (*N*=731). E. Absolute difference of ABI in 4 years (*N*=731). Solid line: adjusted for age and gender, and dotted lines: adjusted for age, gender, SBP, smoking, HDL-cholesterol, total cholesterol, BMI and HbA1c.


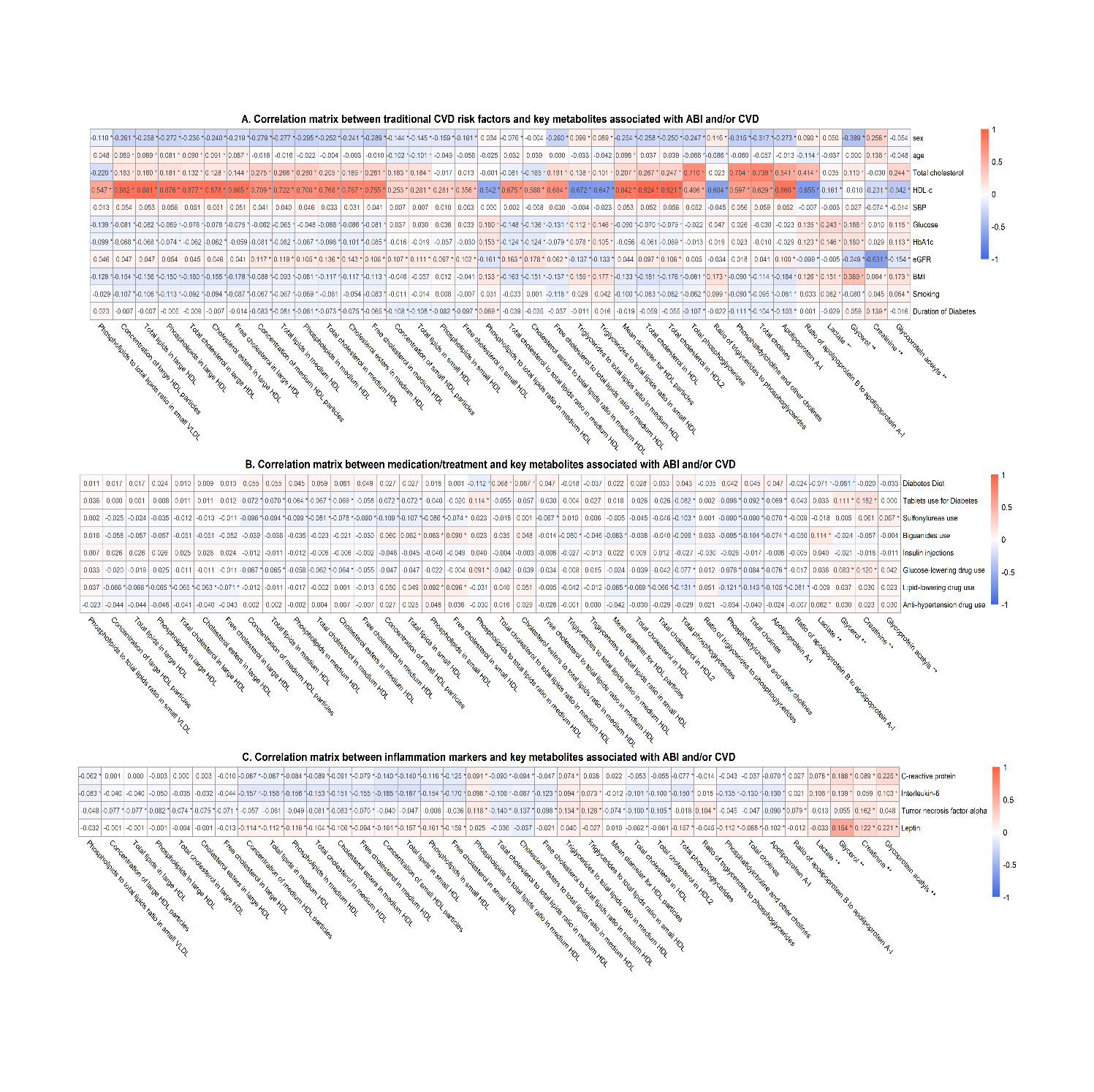


# Figure S4. Correlation matrix between covariates and key metabolites associated with baseline ABI and/or prevalent CVD.

*: statistical significance. †: shared metabolites of baseline ABI and prevalent CVD.

**Table S1. Frequency of constituent endpoints for both prevalent CVD and incident CVD in ET2DS.**

| **CVD endpoints** | **Prevalence** |  | **Incidence*** |
| --- | --- | --- | --- |
| Coronary Interventions | 100 (9.8%) |  | 23 (2.2%) |
| Myocardial infarction | 139 (13.6%) |  | 79 (7.7%) |
| Angina | 285 (27.9%) |  | 53 (5.2%) |
| Stroke | 59 (5.8%) |  | 68 (6.6%) |
| Transient ischemic attack | 30 (2.9%) |  | 11 (1.1%) |
| Peripheral Interventions | 16 (1.6%) |  | 23 (2.2%) |

*: The first event was used where someone had multiple events during the follow-up.

# Table S2. Distribution of serum metabolites of the ET2DS at baseline.

| **Metabolites** | **Units** | **Available Number** | **Number of NULL** | **Number of NA** | **Median** | **Mean** | **SD** |
| --- | --- | --- | --- | --- | --- | --- | --- |
| **VLDL subclasses** |  |  |  |  |  |  |  |
| Concentration of chylomicrons and extremely large VLDL particles | mol/l | 1025 | 0 | 0 | 6.79E-11 | 1.05E-10 | 1.33E-10 |
| Total lipids in chylomicrons and extremely large VLDL | mmol/l | 1025 | 0 | 0 | 1.44E-02 | 2.23E-02 | 2.84E-02 |
| Phospholipids in chylomicrons and extremely large VLDL | mmol/l | 1025 | 0 | 0 | 1.29E-03 | 2.46E-03 | 3.52E-03 |
| Total cholesterol in chylomicrons and extremely large VLDL | mmol/l | 1025 | 0 | 0 | 2.59E-03 | 3.97E-03 | 5.12E-03 |
| Cholesterol esters in chylomicrons and extremely large VLDL | mmol/l | 1025 | 0 | 0 | 1.71E-03 | 2.33E-03 | 2.88E-03 |
| Free cholesterol in chylomicrons and extremely large VLDL | mmol/l | 1025 | 0 | 0 | 8.49E-04 | 1.64E-03 | 2.34E-03 |
| Triglycerides in chylomicrons and extremely large VLDL | mmol/l | 1025 | 0 | 0 | 1.05E-02 | 1.59E-02 | 1.99E-02 |
| Concentration of very large VLDL particles | mol/l | 1025 | 0 | 0 | 5.44E-10 | 7.75E-10 | 8.74E-10 |
| Total lipids in very large VLDL | mmol/l | 1025 | 0 | 0 | 5.12E-02 | 7.40E-02 | 8.44E-02 |
| Phospholipids in very large VLDL | mmol/l | 1025 | 0 | 0 | 7.26E-03 | 1.13E-02 | 1.37E-02 |
| Total cholesterol in very large VLDL | mmol/l | 1025 | 0 | 0 | 7.57E-03 | 1.23E-02 | 1.56E-02 |
| Cholesterol esters in very large VLDL | mmol/l | 1025 | 0 | 0 | 4.68E-03 | 7.10E-03 | 8.51E-03 |
| Free cholesterol in very large VLDL | mmol/l | 1025 | 0 | 0 | 2.90E-03 | 5.18E-03 | 7.16E-03 |
| Triglycerides in very large VLDL | mmol/l | 1025 | 0 | 0 | 3.60E-02 | 5.04E-02 | 5.56E-02 |
| Concentration of large VLDL particles | mol/l | 1025 | 0 | 0 | 5.08E-09 | 6.29E-09 | 4.99E-09 |
| Total lipids in large VLDL | mmol/l | 1025 | 0 | 0 | 2.91E-01 | 3.61E-01 | 2.88E-01 |
| Phospholipids in large VLDL | mmol/l | 1025 | 0 | 0 | 5.26E-02 | 6.59E-02 | 5.21E-02 |
| Total cholesterol in large VLDL | mmol/l | 1025 | 0 | 0 | 6.22E-02 | 7.71E-02 | 6.19E-02 |
| Cholesterol esters in large VLDL | mmol/l | 1025 | 0 | 0 | 3.59E-02 | 4.25E-02 | 2.97E-02 |
| Free cholesterol in large VLDL | mmol/l | 1025 | 0 | 0 | 2.58E-02 | 3.45E-02 | 3.28E-02 |
| Triglycerides in large VLDL | mmol/l | 1025 | 0 | 0 | 1.75E-01 | 2.18E-01 | 1.75E-01 |
| Concentration of medium VLDL particles | mol/l | 1025 | 0 | 0 | 1.92E-08 | 2.17E-08 | 1.25E-08 |
| Total lipids in medium VLDL | mmol/l | 1025 | 0 | 0 | 6.37E-01 | 7.21E-01 | 4.13E-01 |
| Phospholipids in medium VLDL | mmol/l | 1025 | 0 | 0 | 1.29E-01 | 1.44E-01 | 7.76E-02 |
| Total cholesterol in medium VLDL | mmol/l | 1025 | 0 | 0 | 1.65E-01 | 1.85E-01 | 9.61E-02 |
| Cholesterol esters in medium VLDL | mmol/l | 1025 | 0 | 0 | 9.17E-02 | 1.02E-01 | 4.70E-02 |
| Free cholesterol in medium VLDL | mmol/l | 1025 | 0 | 0 | 7.19E-02 | 8.23E-02 | 5.13E-02 |
| Triglycerides in medium VLDL | mmol/l | 1025 | 0 | 0 | 3.40E-01 | 3.92E-01 | 2.43E-01 |
| Concentration of small VLDL particles | mol/l | 1025 | 0 | 0 | 3.22E-08 | 3.40E-08 | 1.26E-08 |
| Total lipids in small VLDL | mmol/l | 1025 | 0 | 0 | 6.18E-01 | 6.54E-01 | 2.36E-01 |
| Phospholipids in small VLDL | mmol/l | 1025 | 0 | 0 | 1.51E-01 | 1.57E-01 | 4.82E-02 |
| Total cholesterol in small VLDL | mmol/l | 1025 | 0 | 0 | 1.92E-01 | 2.00E-01 | 6.68E-02 |
| Cholesterol esters in small VLDL | mmol/l | 1025 | 0 | 0 | 1.06E-01 | 1.11E-01 | 3.96E-02 |
| Free cholesterol in small VLDL | mmol/l | 1025 | 0 | 0 | 8.40E-02 | 8.85E-02 | 3.21E-02 |
| Triglycerides in small VLDL | mmol/l | 1025 | 0 | 0 | 2.77E-01 | 2.97E-01 | 1.32E-01 |
| Concentration of very small VLDL particles | mol/l | 1025 | 0 | 0 | 3.47E-08 | 3.59E-08 | 9.04E-09 |
| Total lipids in very small VLDL | mmol/l | 1025 | 0 | 0 | 4.29E-01 | 4.43E-01 | 1.12E-01 |
| Phospholipids in very small VLDL | mmol/l | 1025 | 0 | 0 | 1.33E-01 | 1.38E-01 | 3.70E-02 |
| Total cholesterol in very small VLDL | mmol/l | 1025 | 0 | 0 | 1.75E-01 | 1.80E-01 | 5.38E-02 |
| Cholesterol esters in very small VLDL | mmol/l | 1025 | 0 | 0 | 1.10E-01 | 1.13E-01 | 3.86E-02 |
| Free cholesterol in very small VLDL | mmol/l | 1025 | 0 | 0 | 6.53E-02 | 6.75E-02 | 1.89E-02 |
| Triglycerides in very small VLDL | mol/l | 1025 | 0 | 0 | 1.18E-01 | 1.24E-01 | 4.24E-02 |
| Phospholipids to total lipids ratio in chylomicrons and extremely large VLDL | % | 1025 | 0 | 0 | 1.13E+01 | 1.49E+01 | 9.37E+00 |
| Total cholesterol to total lipids ratio in chylomicrons and extremely large VLDL | % | 1025 | 0 | 0 | 1.98E+01 | 2.57E+01 | 1.39E+01 |
| Cholesterol esters to total lipids ratio in chylomicrons and extremely large VLDL | % | 1025 | 0 | 0 | 1.32E+01 | 1.21E+01 | 4.97E+00 |
| Free cholesterol to total lipids ratio in chylomicrons and extremely large VLDL | % | 1025 | 0 | 0 | 7.60E+00 | 1.35E+01 | 1.22E+01 |
| Triglycerides to total lipids ratio in chylomicrons and extremely large VLDL | % | 1025 | 0 | 0 | 7.02E+01 | 5.94E+01 | 2.25E+01 |
| Phospholipids to total lipids ratio in very large VLDL | % | 1025 | 0 | 0 | 1.43E+01 | 1.38E+01 | 3.00E+00 |
| Total cholesterol to total lipids ratio in very large VLDL | % | 1025 | 0 | 0 | 1.46E+01 | 1.36E+01 | 6.77E+00 |
| Cholesterol esters to total lipids ratio in very large VLDL | % | 1025 | 0 | 0 | 8.67E+00 | 8.08E+00 | 4.98E+00 |
| Free cholesterol to total lipids ratio in very large VLDL | % | 1025 | 0 | 0 | 5.61E+00 | 5.48E+00 | 2.47E+00 |
| Triglycerides to total lipids ratio in very large VLDL | % | 1025 | 0 | 0 | 7.09E+01 | 7.27E+01 | 9.09E+00 |
| Phospholipids to total lipids ratio in large VLDL | % | 1025 | 0 | 0 | 1.83E+01 | 1.86E+01 | 1.13E+00 |
| Total cholesterol to total lipids ratio in large VLDL | % | 1025 | 0 | 0 | 2.11E+01 | 2.16E+01 | 3.11E+00 |
| Cholesterol esters to total lipids ratio in large VLDL | % | 1025 | 0 | 0 | 1.28E+01 | 1.35E+01 | 3.78E+00 |
| Free cholesterol to total lipids ratio in large VLDL | % | 1025 | 0 | 0 | 8.95E+00 | 8.13E+00 | 2.62E+00 |
| Triglycerides to total lipids ratio in large VLDL | % | 1025 | 0 | 0 | 6.01E+01 | 5.98E+01 | 3.45E+00 |
| Phospholipids to total lipids ratio in medium VLDL | % | 1025 | 0 | 0 | 2.02E+01 | 2.04E+01 | 1.03E+00 |
| Total cholesterol to total lipids ratio in medium VLDL | % | 1025 | 0 | 0 | 2.60E+01 | 2.68E+01 | 4.16E+00 |
| Cholesterol esters to total lipids ratio in medium VLDL | % | 1025 | 0 | 0 | 1.48E+01 | 1.57E+01 | 4.59E+00 |
| Free cholesterol to total lipids ratio in medium VLDL | % | 1025 | 0 | 0 | 1.13E+01 | 1.11E+01 | 9.81E-01 |
| Triglycerides to total lipids ratio in medium VLDL | % | 1025 | 0 | 0 | 5.37E+01 | 5.28E+01 | 5.00E+00 |
| Phospholipids to total lipids ratio in small VLDL | % | 1025 | 0 | 0 | 2.43E+01 | 2.44E+01 | 1.71E+00 |
| Total cholesterol to total lipids ratio in small VLDL | % | 1025 | 0 | 0 | 3.10E+01 | 3.12E+01 | 4.69E+00 |
| Cholesterol esters to total lipids ratio in small VLDL | % | 1025 | 0 | 0 | 1.75E+01 | 1.77E+01 | 4.61E+00 |
| Free cholesterol to total lipids ratio in small VLDL | % | 1025 | 0 | 0 | 1.35E+01 | 1.35E+01 | 6.81E-01 |
| Triglycerides to total lipids ratio in small VLDL | % | 1025 | 0 | 0 | 4.46E+01 | 4.44E+01 | 5.44E+00 |
| Phospholipids to total lipids ratio in very small VLDL | % | 1025 | 0 | 0 | 3.12E+01 | 3.12E+01 | 2.41E+00 |
| Total cholesterol to total lipids ratio in very small VLDL | % | 1025 | 0 | 0 | 4.13E+01 | 4.06E+01 | 6.27E+00 |
| Cholesterol esters to total lipids ratio in very small VLDL | % | 1025 | 0 | 0 | 2.60E+01 | 2.54E+01 | 6.12E+00 |
| Free cholesterol to total lipids ratio in very small VLDL | % | 1025 | 0 | 0 | 1.53E+01 | 1.52E+01 | 1.32E+00 |
| Triglycerides to total lipids ratio in very small VLDL | % | 1025 | 0 | 0 | 2.74E+01 | 2.82E+01 | 7.01E+00 |
| **IDL subclasses** |  |  |  |  |  |  |  |
| Concentration of IDL particles | mol/l | 1025 | 0 | 0 | 8.32E-08 | 8.58E-08 | 2.27E-08 |
| Total lipids in IDL | mmol/l | 1025 | 0 | 0 | 8.25E-01 | 8.52E-01 | 2.33E-01 |
| Phospholipids in IDL | mmol/l | 1025 | 0 | 0 | 2.38E-01 | 2.44E-01 | 6.12E-02 |
| Total cholesterol in IDL | mmol/l | 1025 | 0 | 0 | 4.75E-01 | 4.90E-01 | 1.55E-01 |
| Cholesterol esters in IDL | mmol/l | 1025 | 0 | 0 | 3.31E-01 | 3.44E-01 | 1.09E-01 |
| Free cholesterol in IDL | mmol/l | 1025 | 0 | 0 | 1.43E-01 | 1.46E-01 | 4.83E-02 |
| Triglycerides in IDL | mmol/l | 1025 | 0 | 0 | 1.13E-01 | 1.19E-01 | 3.48E-02 |
| Phospholipids to total lipids ratio in IDL | % | 1025 | 0 | 0 | 2.88E+01 | 2.88E+01 | 1.18E+00 |
| Total cholesterol to total lipids ratio in IDL | % | 1025 | 0 | 0 | 5.75E+01 | 5.68E+01 | 4.24E+00 |
| Cholesterol esters to total lipids ratio in IDL | % | 1025 | 0 | 0 | 4.03E+01 | 3.99E+01 | 3.41E+00 |
| Free cholesterol to total lipids ratio in IDL | % | 1025 | 0 | 0 | 1.73E+01 | 1.69E+01 | 1.94E+00 |
| Triglycerides to total lipids ratio in IDL | % | 1025 | 0 | 0 | 1.36E+01 | 1.44E+01 | 4.25E+00 |
| **LDL subclasses** |  |  |  |  |  |  |  |
| Concentration of large LDL particles | mol/l | 1025 | 0 | 0 | 1.39E-07 | 1.43E-07 | 4.04E-08 |
| Total lipids in large LDL | mmol/l | 1025 | 0 | 0 | 9.79E-01 | 1.01E+00 | 2.89E-01 |
| Phospholipids in large LDL | mmol/l | 1025 | 0 | 0 | 2.64E-01 | 2.70E-01 | 5.95E-02 |
| Total cholesterol in large LDL | mmol/l | 1025 | 0 | 0 | 6.26E-01 | 6.46E-01 | 2.12E-01 |
| Cholesterol esters in large LDL | mmol/l | 1025 | 0 | 0 | 4.44E-01 | 4.59E-01 | 1.60E-01 |
| Free cholesterol in large LDL | mmol/l | 1025 | 0 | 0 | 1.83E-01 | 1.87E-01 | 5.29E-02 |
| Triglycerides in large LDL | mmol/l | 1025 | 0 | 0 | 9.06E-02 | 9.46E-02 | 2.83E-02 |
| Concentration of medium LDL particles | mol/l | 1025 | 0 | 0 | 1.13E-07 | 1.16E-07 | 3.55E-08 |
| Total lipids in medium LDL | mmol/l | 1025 | 0 | 0 | 5.67E-01 | 5.85E-01 | 1.79E-01 |
| Phospholipids in medium LDL | mmol/l | 1025 | 0 | 0 | 1.65E-01 | 1.69E-01 | 3.50E-02 |
| Total cholesterol in medium LDL | mmol/l | 1025 | 0 | 0 | 3.55E-01 | 3.69E-01 | 1.36E-01 |
| Cholesterol esters in medium LDL | mmol/l | 1025 | 0 | 0 | 2.46E-01 | 2.58E-01 | 1.12E-01 |
| Free cholesterol in medium LDL | mmol/l | 1025 | 0 | 0 | 1.09E-01 | 1.11E-01 | 2.49E-02 |
| Triglycerides in medium LDL | mmol/l | 1025 | 0 | 0 | 4.57E-02 | 4.77E-02 | 1.50E-02 |
| Concentration of small LDL particles | mol/l | 1025 | 0 | 0 | 1.30E-07 | 1.34E-07 | 4.03E-08 |
| Total lipids in small LDL | mmol/l | 1025 | 0 | 0 | 3.61E-01 | 3.71E-01 | 1.12E-01 |
| Phospholipids in small LDL | mmol/l | 1025 | 0 | 0 | 1.19E-01 | 1.21E-01 | 2.45E-02 |
| Total cholesterol in small LDL | mmol/l | 1025 | 0 | 0 | 2.11E-01 | 2.18E-01 | 8.33E-02 |
| Cholesterol esters in small LDL | mmol/l | 1025 | 0 | 0 | 1.49E-01 | 1.56E-01 | 6.83E-02 |
| Free cholesterol in small LDL | mmol/l | 1025 | 0 | 0 | 6.08E-02 | 6.21E-02 | 1.57E-02 |
| Triglycerides in small LDL | mmol/l | 1025 | 0 | 0 | 3.00E-02 | 3.16E-02 | 1.13E-02 |
| Phospholipids to total lipids ratio in large LDL | % | 1025 | 0 | 0 | 2.69E+01 | 2.72E+01 | 2.08E+00 |
| Total cholesterol to total lipids ratio in large LDL | % | 1025 | 0 | 0 | 6.37E+01 | 6.32E+01 | 3.41E+00 |
| Cholesterol esters to total lipids ratio in large LDL | % | 1025 | 0 | 0 | 4.51E+01 | 4.47E+01 | 3.34E+00 |
| Free cholesterol to total lipids ratio in large LDL | % | 1025 | 0 | 0 | 1.87E+01 | 1.85E+01 | 1.44E+00 |
| Triglycerides to total lipids ratio in large LDL | % | 1025 | 0 | 0 | 9.19E+00 | 9.58E+00 | 2.33E+00 |
| Phospholipids to total lipids ratio in medium LDL | % | 1025 | 0 | 0 | 2.90E+01 | 2.98E+01 | 4.07E+00 |
| Total cholesterol to total lipids ratio in medium LDL | % | 1025 | 0 | 0 | 6.28E+01 | 6.18E+01 | 5.17E+00 |
| Cholesterol esters to total lipids ratio in medium LDL | % | 1025 | 0 | 0 | 4.34E+01 | 4.24E+01 | 6.79E+00 |
| Free cholesterol to total lipids ratio in medium LDL | % | 1025 | 0 | 0 | 1.92E+01 | 1.95E+01 | 2.05E+00 |
| Triglycerides to total lipids ratio in medium LDL | % | 1025 | 0 | 0 | 8.00E+00 | 8.37E+00 | 2.13E+00 |
| Phospholipids to total lipids ratio in small LDL | % | 1025 | 0 | 0 | 3.31E+01 | 3.39E+01 | 5.02E+00 |
| Total cholesterol to total lipids ratio in small LDL | % | 1025 | 0 | 0 | 5.86E+01 | 5.74E+01 | 6.41E+00 |
| Cholesterol esters to total lipids ratio in small LDL | % | 1025 | 0 | 0 | 4.16E+01 | 4.03E+01 | 7.40E+00 |
| Free cholesterol to total lipids ratio in small LDL | % | 1025 | 0 | 0 | 1.69E+01 | 1.70E+01 | 1.52E+00 |
| Triglycerides to total lipids ratio in small LDL | % | 1025 | 0 | 0 | 8.21E+00 | 8.74E+00 | 2.61E+00 |
| **HDL subclasses** |  |  |  |  |  |  |  |
| Concentration of very large HDL particles | mol/l | 1025 | 0 | 0 | 1.80E-07 | 2.03E-07 | 1.99E-07 |
| Total lipids in very large HDL | mmol/l | 1025 | 0 | 0 | 1.79E-01 | 2.02E-01 | 2.04E-01 |
| Phospholipids in very large HDL | mmol/l | 1025 | 0 | 0 | 1.23E-01 | 1.27E-01 | 1.22E-01 |
| Total cholesterol in very large HDL | mmol/l | 1025 | 0 | 0 | 4.94E-02 | 6.95E-02 | 8.08E-02 |
| Cholesterol esters in very large HDL | mmol/l | 1025 | 0 | 0 | 2.10E-02 | 3.98E-02 | 5.34E-02 |
| Free cholesterol in very large HDL | mmol/l | 1025 | 0 | 0 | 2.85E-02 | 2.97E-02 | 2.86E-02 |
| Triglycerides in very large HDL | mmol/l | 1025 | 0 | 0 | 2.54E-03 | 5.07E-03 | 6.56E-03 |
| Concentration of large HDL particles | mol/l | 1025 | 0 | 0 | 6.81E-07 | 7.37E-07 | 5.11E-07 |
| Total lipids in large HDL | mmol/l | 1025 | 0 | 0 | 4.19E-01 | 4.56E-01 | 3.27E-01 |
| Phospholipids in large HDL | mmol/l | 1025 | 0 | 0 | 2.41E-01 | 2.48E-01 | 1.54E-01 |
| Total cholesterol in large HDL | mmol/l | 1025 | 0 | 0 | 1.64E-01 | 1.94E-01 | 1.65E-01 |
| Cholesterol esters in large HDL | mmol/l | 1025 | 0 | 0 | 1.32E-01 | 1.55E-01 | 1.27E-01 |
| Free cholesterol in large HDL | mmol/l | 1025 | 0 | 0 | 3.10E-02 | 3.92E-02 | 3.79E-02 |
| Triglycerides in large HDL | mmol/l | 1025 | 0 | 0 | 1.20E-02 | 1.43E-02 | 1.37E-02 |
| Concentration of medium HDL particles | mol/l | 1025 | 0 | 0 | 2.11E-06 | 2.14E-06 | 4.47E-07 |
| Total lipids in medium HDL | mmol/l | 1025 | 0 | 0 | 8.89E-01 | 8.99E-01 | 1.94E-01 |
| Phospholipids in medium HDL | mmol/l | 1025 | 0 | 0 | 4.16E-01 | 4.21E-01 | 8.36E-02 |
| Total cholesterol in medium HDL | mmol/l | 1025 | 0 | 0 | 4.18E-01 | 4.22E-01 | 1.09E-01 |
| Cholesterol esters in medium HDL | mmol/l | 1025 | 0 | 0 | 3.39E-01 | 3.43E-01 | 8.62E-02 |
| Free cholesterol in medium HDL | mmol/l | 1025 | 0 | 0 | 7.83E-02 | 7.93E-02 | 2.29E-02 |
| Triglycerides in medium HDL | mmol/l | 1025 | 0 | 0 | 5.53E-02 | 5.63E-02 | 1.29E-02 |
| Concentration of small HDL particles | mol/l | 1025 | 0 | 0 | 5.45E-06 | 5.47E-06 | 5.62E-07 |
| Total lipids in small HDL | mmol/l | 1025 | 0 | 0 | 1.21E+00 | 1.21E+00 | 1.26E-01 |
| Phospholipids in small HDL | mmol/l | 1025 | 0 | 0 | 6.20E-01 | 6.23E-01 | 8.18E-02 |
| Total cholesterol in small HDL | mmol/l | 1025 | 0 | 0 | 5.32E-01 | 5.29E-01 | 6.62E-02 |
| Cholesterol esters in small HDL | mmol/l | 1025 | 0 | 0 | 4.15E-01 | 4.11E-01 | 5.84E-02 |
| Free cholesterol in small HDL | mmol/l | 1025 | 0 | 0 | 1.16E-01 | 1.18E-01 | 1.55E-02 |
| Triglycerides in small HDL | mmol/l | 1025 | 0 | 0 | 6.07E-02 | 6.14E-02 | 1.41E-02 |
| Phospholipids to total lipids ratio in very large HDL | % | 1025 | 0 | 0 | 5.97E+01 | 5.03E+01 | 2.20E+01 |
| Total cholesterol to total lipids ratio in very large HDL | % | 1025 | 0 | 0 | 3.77E+01 | 4.45E+01 | 1.92E+01 |
| Cholesterol esters to total lipids ratio in very large HDL | % | 1025 | 0 | 0 | 1.13E+01 | 1.40E+01 | 7.80E+00 |
| Free cholesterol to total lipids ratio in very large HDL | % | 1025 | 0 | 0 | 1.63E+01 | 3.05E+01 | 2.23E+01 |
| Triglycerides to total lipids ratio in very large HDL | % | 1025 | 0 | 0 | 3.97E+00 | 5.28E+00 | 4.66E+00 |
| Phospholipids to total lipids ratio in large HDL | % | 1025 | 0 | 0 | 5.73E+01 | 5.96E+01 | 8.80E+00 |
| Total cholesterol to total lipids ratio in large HDL | % | 1025 | 0 | 0 | 3.95E+01 | 3.77E+01 | 8.14E+00 |
| Cholesterol esters to total lipids ratio in large HDL | % | 1025 | 0 | 0 | 3.21E+01 | 3.10E+01 | 5.14E+00 |
| Free cholesterol to total lipids ratio in large HDL | % | 1025 | 0 | 0 | 7.54E+00 | 6.67E+00 | 3.17E+00 |
| Triglycerides to total lipids ratio in large HDL | % | 1025 | 0 | 0 | 2.49E+00 | 2.70E+00 | 2.04E+00 |
| Phospholipids to total lipids ratio in medium HDL | % | 1025 | 0 | 0 | 4.68E+01 | 4.70E+01 | 1.52E+00 |
| Total cholesterol to total lipids ratio in medium HDL | % | 1025 | 0 | 0 | 4.69E+01 | 4.65E+01 | 2.97E+00 |
| Cholesterol esters to total lipids ratio in medium HDL | % | 1025 | 0 | 0 | 3.81E+01 | 3.78E+01 | 2.44E+00 |
| Free cholesterol to total lipids ratio in medium HDL | % | 1025 | 0 | 0 | 8.85E+00 | 8.68E+00 | 8.37E-01 |
| Triglycerides to total lipids ratio in medium HDL | % | 1025 | 0 | 0 | 6.25E+00 | 6.46E+00 | 1.76E+00 |
| Phospholipids to total lipids ratio in small HDL | % | 1025 | 0 | 0 | 5.15E+01 | 5.13E+01 | 3.28E+00 |
| Total cholesterol to total lipids ratio in small HDL | % | 1025 | 0 | 0 | 4.36E+01 | 4.36E+01 | 3.54E+00 |
| Cholesterol esters to total lipids ratio in small HDL | % | 1025 | 0 | 0 | 3.39E+01 | 3.39E+01 | 3.85E+00 |
| Free cholesterol to total lipids ratio in small HDL | % | 1025 | 0 | 0 | 9.69E+00 | 9.67E+00 | 5.12E-01 |
| Triglycerides to total lipids ratio in small HDL | % | 1025 | 0 | 0 | 4.97E+00 | 5.10E+00 | 1.27E+00 |
| **Lipoprotein particle sizes** |  |  |  |  |  |  |  |
| Mean diameter for VLDL particles | nm | 1025 | 0 | 0 | 3.74E+01 | 3.74E+01 | 1.48E+00 |
| Mean diameter for LDL particles | nm | 1025 | 0 | 0 | 2.36E+01 | 2.36E+01 | 1.97E-01 |
| Mean diameter for HDL particles | nm | 1025 | 0 | 0 | 9.65E+00 | 9.68E+00 | 2.49E-01 |
| Serum total cholesterol | mmol/l | 1025 | 0 | 0 | 3.53E+00 | 3.61E+00 | 7.94E-01 |
| Total cholesterol in VLDL | mmol/l | 1025 | 0 | 0 | 6.13E-01 | 6.59E-01 | 2.47E-01 |
| Remnant cholesterol (non-HDL, non-LDL -cholesterol) | mmol/l | 1025 | 0 | 0 | 1.09E+00 | 1.15E+00 | 3.33E-01 |
| Total cholesterol in LDL | mmol/l | 1025 | 0 | 0 | 1.19E+00 | 1.23E+00 | 4.28E-01 |
| Total cholesterol in HDL | mmol/l | 1025 | 0 | 0 | 1.18E+00 | 1.22E+00 | 3.26E-01 |
| Total cholesterol in HDL2 | mmol/l | 1025 | 1 | 0 | 7.34E-01 | 7.69E-01 | 3.09E-01 |
| Total cholesterol in HDL3 | mmol/l | 1025 | 0 | 0 | 4.52E-01 | 4.54E-01 | 3.04E-02 |
| Esterified cholesterol | mmol/l | 1024 | 0 | 1 | 2.51E+00 | 2.57E+00 | 5.81E-01 |
| Free cholesterol | mmol/l | 1024 | 0 | 1 | 1.01E+00 | 1.04E+00 | 2.27E-01 |
| Serum total triglycerides | mmol/l | 1025 | 0 | 0 | 1.39E+00 | 1.53E+00 | 7.17E-01 |
| Triglycerides in VLDL | mmol/l | 1025 | 0 | 0 | 9.69E-01 | 1.10E+00 | 6.49E-01 |
| Triglycerides in LDL | mmol/l | 1025 | 0 | 0 | 1.67E-01 | 1.74E-01 | 5.34E-02 |
| Triglycerides in HDL | mmol/l | 1025 | 0 | 0 | 1.36E-01 | 1.41E-01 | 3.37E-02 |
| Total phosphoglycerides | mmol/l | 1024 | 0 | 1 | 1.66E+00 | 1.69E+00 | 3.33E-01 |
| Ratio of triglycerides to phosphoglycerides |  | 1024 | 0 | 1 | 6.80E-01 | 7.32E-01 | 3.19E-01 |
| Phosphatidylcholine and other cholines | mmol/l | 1024 | 0 | 1 | 1.77E+00 | 1.79E+00 | 3.09E-01 |
| Sphingomyelins | mmol/l | 1024 | 0 | 1 | 4.03E-01 | 4.10E-01 | 7.27E-02 |
| Total cholines | mmol/l | 1024 | 0 | 1 | 2.09E+00 | 2.12E+00 | 3.39E-01 |
| **Apolipoproteins** |  |  |  |  |  |  |  |
| Apolipoprotein A-I | g/l | 1025 | 0 | 0 | 1.36E+00 | 1.38E+00 | 1.91E-01 |
| Apolipoprotein B | g/l | 1025 | 0 | 0 | 7.62E-01 | 7.94E-01 | 1.86E-01 |
| Ratio of apolipoprotein B to apolipoprotein A-I |  | 1025 | 0 | 0 | 5.68E-01 | 5.83E-01 | 1.42E-01 |
| **Fatty acids** |  |  |  |  |  |  |  |
| Total fatty acids | mmol/l | 1024 | 0 | 1 | 9.55E+00 | 9.90E+00 | 2.25E+00 |
| Estimated degree of unsaturation |  | 1024 | 0 | 1 | 1.22E+00 | 1.23E+00 | 9.35E-02 |
| 22:6, docosahexaenoic acid | mmol/l | 1024 | 0 | 1 | 1.88E-01 | 1.95E-01 | 6.03E-02 |
| 18:2, linoleic acid | mmol/l | 1024 | 0 | 1 | 2.47E+00 | 2.52E+00 | 5.24E-01 |
| Omega-3 fatty acids | mmol/l | 1024 | 0 | 1 | 5.45E-01 | 5.69E-01 | 1.45E-01 |
| Omega-6 fatty acids | mmol/l | 1024 | 0 | 1 | 3.10E+00 | 3.14E+00 | 6.08E-01 |
| Polyunsaturated fatty acids | mmol/l | 1024 | 0 | 1 | 3.67E+00 | 3.71E+00 | 7.00E-01 |
| Monounsaturated fatty acids; 16:1, 18:1 | mmol/l | 1024 | 0 | 1 | 2.44E+00 | 2.60E+00 | 9.10E-01 |
| Saturated fatty acids | mmol/l | 1024 | 0 | 1 | 3.46E+00 | 3.60E+00 | 9.20E-01 |
| Ratio of 22:6 docosahexaenoic acid to total fatty acids | % | 1024 | 0 | 1 | 1.94E+00 | 2.00E+00 | 5.73E-01 |
| Ratio of 18:2 linoleic acid to total fatty acids | % | 1024 | 0 | 1 | 2.55E+01 | 2.58E+01 | 4.04E+00 |
| Ratio of omega-3 fatty acids to total fatty acids | % | 1024 | 0 | 1 | 5.71E+00 | 5.84E+00 | 1.28E+00 |
| Ratio of omega-6 fatty acids to total fatty acids | % | 1024 | 0 | 1 | 3.19E+01 | 3.21E+01 | 4.47E+00 |
| Ratio of polyunsaturated fatty acids to total fatty acids | % | 1024 | 0 | 1 | 3.77E+01 | 3.80E+01 | 5.06E+00 |
| Ratio of monounsaturated fatty acids to total fatty acids | % | 1024 | 0 | 1 | 2.59E+01 | 2.58E+01 | 4.44E+00 |
| Ratio of saturated fatty acids to total fatty acids | % | 1024 | 0 | 1 | 3.63E+01 | 3.63E+01 | 2.57E+00 |
| **Glycolysis related metabolites** |  |  |  |  |  |  |  |
| Glucose | mmol/l | 1024 | 0 | 1 | 6.23E+00 | 6.50E+00 | 1.68E+00 |
| Lactate | mmol/l | 1025 | 0 | 0 | 1.36E+00 | 1.44E+00 | 4.39E-01 |
| Pyruvate | mmol/l | 1006 | 0 | 19 | 6.30E-02 | 7.12E-02 | 3.58E-02 |
| Citrate | mmol/l | 1025 | 0 | 0 | 1.37E-01 | 1.38E-01 | 2.29E-02 |
| Glycerol | mmol/l | 1022 | 0 | 3 | 8.99E-02 | 9.48E-02 | 2.98E-02 |
| **Amino acid** |  |  |  |  |  |  |  |
| Alanine | mmol/l | 1025 | 0 | 0 | 3.45E-01 | 3.50E-01 | 5.71E-02 |
| Glutamine | mmol/l | 1025 | 0 | 0 | 4.33E-01 | 4.35E-01 | 6.17E-02 |
| Glycine | mmol/l | 1025 | 0 | 0 | 2.49E-01 | 2.56E-01 | 4.37E-02 |
| Histidine | mmol/l | 1024 | 0 | 1 | 5.22E-02 | 5.23E-02 | 9.09E-03 |
| Isoleucine | mmol/l | 1025 | 0 | 0 | 7.14E-02 | 7.32E-02 | 1.67E-02 |
| Leucine | mmol/l | 1025 | 0 | 0 | 7.71E-02 | 7.86E-02 | 1.65E-02 |
| Valine | mmol/l | 1025 | 0 | 0 | 1.79E-01 | 1.80E-01 | 3.33E-02 |
| Phenylalanine | mmol/l | 1025 | 0 | 0 | 8.01E-02 | 8.15E-02 | 1.17E-02 |
| Tyrosine | mmol/l | 1024 | 0 | 1 | 5.35E-02 | 5.52E-02 | 1.35E-02 |
| **Ketone Bodies** |  |  |  |  |  |  |  |
| Acetate | mmol/l | 1025 | 0 | 0 | 3.71E-02 | 3.89E-02 | 1.07E-02 |
| Acetoacetate | mmol/l | 1025 | 0 | 0 | 4.03E-02 | 4.71E-02 | 2.64E-02 |
| 3-hydroxybutyrate | mmol/l | 1022 | 0 | 3 | 1.20E-01 | 1.47E-01 | 9.62E-02 |
| **Fluid Balance** |  |  |  |  |  |  |  |
| Creatinine | mmol/l | 1024 | 0 | 1 | 6.35E-02 | 6.79E-02 | 2.18E-02 |
| Albumin | signal area | 1025 | 0 | 0 | 9.45E-02 | 9.46E-02 | 5.98E-03 |
| **Inflammation** |  |  |  |  |  |  |  |
| Glycoprotein acetyls | mmol/l | 1025 | 0 | 0 | 1.38E+00 | 1.42E+00 | 2.83E-01 |

Null: Very low concentration (below detection limit); NA: Value was rejected by automatic sample and measurement quality control.

# Table S3. Association between each metabolite and baseline ABI, adjusted for age and sex.

| **Metabolites** | **Beta** | **95% CI** | ***p* value** |
| --- | --- | --- | --- |
| **VLDL** |  |  |  |
| Concentration of chylomicrons and extremely large VLDL particles | -1.30E-02 | (-2.37E-02, -2.34E-03) | 1.71E-02 |
| Total lipids in chylomicrons and extremely large VLDL | -1.31E-02 | (-2.38E-02, -2.40E-03) | 1.66E-02 |
| Phospholipids in chylomicrons and extremely large VLDL | -1.23E-02 | (-2.30E-02, -1.63E-03) | 2.42E-02 |
| Total cholesterol in chylomicrons and extremely large VLDL | -1.40E-02 | (-2.47E-02, -3.34E-03) | 1.02E-02 |
| Cholesterol esters in chylomicrons and extremely large VLDL | -1.42E-02 | (-2.49E-02, -3.51E-03) | 9.31E-03 |
| Free cholesterol in chylomicrons and extremely large VLDL | -1.32E-02 | (-2.39E-02, -2.49E-03) | 1.59E-02 |
| Triglycerides in chylomicrons and extremely large VLDL | -1.29E-02 | (-2.36E-02, -2.21E-03) | 1.82E-02 |
| Concentration of very large VLDL particles | -1.41E-02 | (-2.48E-02, -3.35E-03) | 1.02E-02 |
| Total lipids in very large VLDL | -1.41E-02 | (-2.48E-02, -3.38E-03) | 1.01E-02 |
| Phospholipids in very large VLDL | -1.38E-02 | (-2.45E-02, -3.07E-03) | 1.18E-02 |
| Total cholesterol in very large VLDL | -1.45E-02 | (-2.52E-02, -3.79E-03) | 8.06E-03 |
| Cholesterol esters in very large VLDL | -1.49E-02 | (-2.56E-02, -4.20E-03) | 6.43E-03 |
| Free cholesterol in very large VLDL | -1.39E-02 | (-2.46E-02, -3.19E-03) | 1.11E-02 |
| Triglycerides in very large VLDL | -1.39E-02 | (-2.46E-02, -3.21E-03) | 1.10E-02 |
| Concentration of large VLDL particles | -1.34E-02 | (-2.42E-02, -2.71E-03) | 1.43E-02 |
| Total lipids in large VLDL | -1.35E-02 | (-2.42E-02, -2.73E-03) | 1.41E-02 |
| Phospholipids in large VLDL | -1.34E-02 | (-2.42E-02, -2.70E-03) | 1.43E-02 |
| Total cholesterol in large VLDL | -1.39E-02 | (-2.46E-02, -3.14E-03) | 1.14E-02 |
| Cholesterol esters in large VLDL | -1.41E-02 | (-2.48E-02, -3.37E-03) | 1.01E-02 |
| Free cholesterol in large VLDL | -1.34E-02 | (-2.41E-02, -2.70E-03) | 1.43E-02 |
| Triglycerides in large VLDL | -1.32E-02 | (-2.40E-02, -2.50E-03) | 1.58E-02 |
| Concentration of medium VLDL particles | -1.37E-02 | (-2.44E-02, -2.95E-03) | 1.27E-02 |
| Total lipids in medium VLDL | -1.37E-02 | (-2.45E-02, -3.00E-03) | 1.23E-02 |
| Phospholipids in medium VLDL | -1.36E-02 | (-2.43E-02, -2.84E-03) | 1.34E-02 |
| Total cholesterol in medium VLDL | -1.41E-02 | (-2.48E-02, -3.37E-03) | 1.01E-02 |
| Cholesterol esters in medium VLDL | -1.39E-02 | (-2.46E-02, -3.20E-03) | 1.10E-02 |
| Free cholesterol in medium VLDL | -1.36E-02 | (-2.44E-02, -2.92E-03) | 1.28E-02 |
| Triglycerides in medium VLDL | -1.35E-02 | (-2.42E-02, -2.71E-03) | 1.43E-02 |
| Concentration of small VLDL particles | -1.20E-02 | (-2.28E-02, -1.29E-03) | 2.83E-02 |
| Total lipids in small VLDL | -1.18E-02 | (-2.25E-02, -1.03E-03) | 3.19E-02 |
| Phospholipids in small VLDL | -9.74E-03 | (-2.05E-02, 1.00E-03) | 7.58E-02 |
| Total cholesterol in small VLDL | -9.53E-03 | (-2.03E-02, 1.23E-03) | 8.27E-02 |
| Cholesterol esters in small VLDL | -7.82E-03 | (-1.86E-02, 2.95E-03) | 1.55E-01 |
| Free cholesterol in small VLDL | -1.02E-02 | (-2.09E-02, 5.77E-04) | 6.39E-02 |
| Triglycerides in small VLDL | -1.27E-02 | (-2.35E-02, -1.98E-03) | 2.04E-02 |
| Concentration of very small VLDL particles | -1.14E-02 | (-2.24E-02, -3.75E-04) | 4.30E-02 |
| Total lipids in very small VLDL | -1.08E-02 | (-2.18E-02, 2.69E-04) | 5.61E-02 |
| Phospholipids in very small VLDL | -6.81E-03 | (-1.79E-02, 4.24E-03) | 2.27E-01 |
| Total cholesterol in very small VLDL | -7.67E-03 | (-1.88E-02, 3.50E-03) | 1.79E-01 |
| Cholesterol esters in very small VLDL | -6.93E-03 | (-1.81E-02, 4.21E-03) | 2.23E-01 |
| Free cholesterol in very small VLDL | -7.50E-03 | (-1.86E-02, 3.58E-03) | 1.85E-01 |
| Triglycerides in very small VLDL | -1.23E-02 | (-2.31E-02, -1.56E-03) | 2.50E-02 |
| Phospholipids to total lipids ratio in chylomicrons and extremely large VLDL | 7.15E-03 | (-3.55E-03, 1.79E-02) | 1.90E-01 |
| Total cholesterol to total lipids ratio in chylomicrons and extremely large VLDL | 6.86E-03 | (-3.85E-03, 1.76E-02) | 2.10E-01 |
| Cholesterol esters to total lipids ratio in chylomicrons and extremely large VLDL | -7.20E-04 | (-1.15E-02, 1.00E-02) | 8.96E-01 |
| Free cholesterol to total lipids ratio in chylomicrons and extremely large VLDL | 8.07E-03 | (-2.64E-03, 1.88E-02) | 1.40E-01 |
| Triglycerides to total lipids ratio in chylomicrons and extremely large VLDL | -7.20E-03 | (-1.79E-02, 3.50E-03) | 1.87E-01 |
| Phospholipids to total lipids ratio in very large VLDL | -8.18E-03 | (-1.90E-02, 2.61E-03) | 1.38E-01 |
| Total cholesterol to total lipids ratio in very large VLDL | -6.71E-03 | (-1.74E-02, 3.99E-03) | 2.19E-01 |
| Cholesterol esters to total lipids ratio in very large VLDL | -4.15E-03 | (-1.48E-02, 6.55E-03) | 4.47E-01 |
| Free cholesterol to total lipids ratio in very large VLDL | -1.01E-02 | (-2.08E-02, 6.27E-04) | 6.53E-02 |
| Triglycerides to total lipids ratio in very large VLDL | 7.68E-03 | (-3.05E-03, 1.84E-02) | 1.61E-01 |
| Phospholipids to total lipids ratio in large VLDL | 1.64E-03 | (-9.11E-03, 1.24E-02) | 7.65E-01 |
| Total cholesterol to total lipids ratio in large VLDL | -2.32E-03 | (-1.31E-02, 8.44E-03) | 6.72E-01 |
| Cholesterol esters to total lipids ratio in large VLDL | 2.95E-03 | (-7.78E-03, 1.37E-02) | 5.90E-01 |
| Free cholesterol to total lipids ratio in large VLDL | -6.99E-03 | (-1.77E-02, 3.73E-03) | 2.01E-01 |
| Triglycerides to total lipids ratio in large VLDL | 1.57E-03 | (-9.22E-03, 1.24E-02) | 7.75E-01 |
| Phospholipids to total lipids ratio in medium VLDL | 1.00E-02 | (-7.30E-04, 2.08E-02) | 6.80E-02 |
| Total cholesterol to total lipids ratio in medium VLDL | 3.26E-03 | (-7.61E-03, 1.41E-02) | 5.57E-01 |
| Cholesterol esters to total lipids ratio in medium VLDL | 4.44E-03 | (-6.36E-03, 1.52E-02) | 4.20E-01 |
| Free cholesterol to total lipids ratio in medium VLDL | -7.06E-03 | (-1.78E-02, 3.69E-03) | 1.99E-01 |
| Triglycerides to total lipids ratio in medium VLDL | -4.81E-03 | (-1.57E-02, 6.05E-03) | 3.85E-01 |
| Phospholipids to total lipids ratio in small VLDL | 1.45E-02 | (3.79E-03, 2.52E-02) | 8.14E-03 |
| Total cholesterol to total lipids ratio in small VLDL | 7.83E-03 | (-3.01E-03, 1.87E-02) | 1.57E-01 |
| Cholesterol esters to total lipids ratio in small VLDL | 6.35E-03 | (-4.45E-03, 1.71E-02) | 2.50E-01 |
| Free cholesterol to total lipids ratio in small VLDL | 1.08E-02 | (-1.60E-04, 2.17E-02) | 5.37E-02 |
| Triglycerides to total lipids ratio in small VLDL | -1.14E-02 | (-2.23E-02, -5.86E-04) | 3.91E-02 |
| Phospholipids to total lipids ratio in very small VLDL | 1.20E-02 | (1.29E-03, 2.27E-02) | 2.82E-02 |
| Total cholesterol to total lipids ratio in very small VLDL | 2.13E-03 | (-8.76E-03, 1.30E-02) | 7.01E-01 |
| Cholesterol esters to total lipids ratio in very small VLDL | 5.10E-04 | (-1.03E-02, 1.14E-02) | 9.27E-01 |
| Free cholesterol to total lipids ratio in very small VLDL | 7.47E-03 | (-3.26E-03, 1.82E-02) | 1.73E-01 |
| Triglycerides to total lipids ratio in very small VLDL | -6.09E-03 | (-1.69E-02, 4.75E-03) | 2.71E-01 |
| Concentration of IDL particles | -2.39E-03 | (-1.34E-02, 8.63E-03) | 6.71E-01 |
| **IDL** |  |  |  |
| Total lipids in IDL | -1.51E-03 | (-1.25E-02, 9.49E-03) | 7.88E-01 |
| Phospholipids in IDL | 4.55E-04 | (-1.05E-02, 1.14E-02) | 9.35E-01 |
| Total cholesterol in IDL | 1.55E-04 | (-1.08E-02, 1.11E-02) | 9.78E-01 |
| Cholesterol esters in IDL | -7.17E-04 | (-1.17E-02, 1.02E-02) | 8.98E-01 |
| Free cholesterol in IDL | 2.11E-03 | (-8.85E-03, 1.31E-02) | 7.06E-01 |
| Triglycerides in IDL | -1.15E-02 | (-2.24E-02, -5.59E-04) | 3.96E-02 |
| Phospholipids to total lipids ratio in IDL | 1.35E-02 | (2.63E-03, 2.43E-02) | 1.51E-02 |
| Total cholesterol to total lipids ratio in IDL | 9.28E-03 | (-1.43E-03, 2.00E-02) | 8.96E-02 |
| Cholesterol esters to total lipids ratio in IDL | 4.53E-03 | (-6.17E-03, 1.52E-02) | 4.07E-01 |
| Free cholesterol to total lipids ratio in IDL | 1.24E-02 | (1.62E-03, 2.31E-02) | 2.44E-02 |
| Triglycerides to total lipids ratio in IDL | -1.28E-02 | (-2.35E-02, -2.15E-03) | 1.87E-02 |
| **LDL** |  |  |  |
| Concentration of large LDL particles | 5.47E-04 | (-1.03E-02, 1.14E-02) | 9.22E-01 |
| Total lipids in large LDL | 9.92E-04 | (-9.89E-03, 1.19E-02) | 8.58E-01 |
| Phospholipids in large LDL | 1.37E-03 | (-9.53E-03, 1.23E-02) | 8.05E-01 |
| Total cholesterol in large LDL | 1.93E-03 | (-8.91E-03, 1.28E-02) | 7.27E-01 |
| Cholesterol esters in large LDL | 1.32E-03 | (-9.51E-03, 1.22E-02) | 8.11E-01 |
| Free cholesterol in large LDL | 3.72E-03 | (-7.14E-03, 1.46E-02) | 5.02E-01 |
| Triglycerides in large LDL | -7.56E-03 | (-1.86E-02, 3.49E-03) | 1.80E-01 |
| Concentration of medium LDL particles | 1.31E-03 | (-9.52E-03, 1.21E-02) | 8.12E-01 |
| Total lipids in medium LDL | 1.64E-03 | (-9.18E-03, 1.25E-02) | 7.67E-01 |
| Phospholipids in medium LDL | -3.79E-04 | (-1.12E-02, 1.05E-02) | 9.46E-01 |
| Total cholesterol in medium LDL | 2.71E-03 | (-8.08E-03, 1.35E-02) | 6.22E-01 |
| Cholesterol esters in medium LDL | 2.69E-03 | (-8.09E-03, 1.35E-02) | 6.25E-01 |
| Free cholesterol in medium LDL | 2.74E-03 | (-8.09E-03, 1.36E-02) | 6.20E-01 |
| Triglycerides in medium LDL | -4.37E-03 | (-1.53E-02, 6.62E-03) | 4.36E-01 |
| Concentration of small LDL particles | 1.14E-03 | (-9.70E-03, 1.20E-02) | 8.37E-01 |
| Total lipids in small LDL | 1.56E-03 | (-9.26E-03, 1.24E-02) | 7.77E-01 |
| Phospholipids in small LDL | -4.73E-04 | (-1.14E-02, 1.04E-02) | 9.32E-01 |
| Total cholesterol in small LDL | 3.25E-03 | (-7.53E-03, 1.40E-02) | 5.55E-01 |
| Cholesterol esters in small LDL | 3.38E-03 | (-7.40E-03, 1.42E-02) | 5.38E-01 |
| Free cholesterol in small LDL | 2.54E-03 | (-8.28E-03, 1.34E-02) | 6.45E-01 |
| Triglycerides in small LDL | -7.55E-03 | (-1.84E-02, 3.26E-03) | 1.72E-01 |
| Phospholipids to total lipids ratio in large LDL | -3.24E-03 | (-1.40E-02, 7.55E-03) | 5.56E-01 |
| Total cholesterol to total lipids ratio in large LDL | 1.03E-02 | (-4.39E-04, 2.09E-02) | 6.05E-02 |
| Cholesterol esters to total lipids ratio in large LDL | 6.08E-03 | (-4.66E-03, 1.68E-02) | 2.67E-01 |
| Free cholesterol to total lipids ratio in large LDL | 1.02E-02 | (-4.69E-04, 2.10E-02) | 6.12E-02 |
| Triglycerides to total lipids ratio in large LDL | -1.23E-02 | (-2.30E-02, -1.56E-03) | 2.50E-02 |
| Phospholipids to total lipids ratio in medium LDL | -7.66E-03 | (-1.84E-02, 3.06E-03) | 1.62E-01 |
| Total cholesterol to total lipids ratio in medium LDL | 9.56E-03 | (-1.14E-03, 2.03E-02) | 8.03E-02 |
| Cholesterol esters to total lipids ratio in medium LDL | 7.84E-03 | (-2.88E-03, 1.86E-02) | 1.52E-01 |
| Free cholesterol to total lipids ratio in medium LDL | -1.80E-03 | (-1.26E-02, 8.97E-03) | 7.43E-01 |
| Triglycerides to total lipids ratio in medium LDL | -8.76E-03 | (-1.96E-02, 2.05E-03) | 1.12E-01 |
| Phospholipids to total lipids ratio in small LDL | -5.31E-03 | (-1.60E-02, 5.40E-03) | 3.32E-01 |
| Total cholesterol to total lipids ratio in small LDL | 9.21E-03 | (-1.49E-03, 1.99E-02) | 9.19E-02 |
| Cholesterol esters to total lipids ratio in small LDL | 7.58E-03 | (-3.13E-03, 1.83E-02) | 1.66E-01 |
| Free cholesterol to total lipids ratio in small LDL | 2.01E-03 | (-8.73E-03, 1.28E-02) | 7.14E-01 |
| Triglycerides to total lipids ratio in small LDL | -1.24E-02 | (-2.30E-02, -1.68E-03) | 2.35E-02 |
| **HDL** |  |  |  |
| Concentration of very large HDL particles | 5.28E-03 | (-5.66E-03, 1.62E-02) | 3.44E-01 |
| Total lipids in very large HDL | 5.06E-03 | (-5.88E-03, 1.60E-02) | 3.65E-01 |
| Phospholipids in very large HDL | 6.86E-03 | (-4.11E-03, 1.78E-02) | 2.21E-01 |
| Total cholesterol in very large HDL | 2.82E-03 | (-8.06E-03, 1.37E-02) | 6.12E-01 |
| Cholesterol esters in very large HDL | 2.85E-03 | (-7.98E-03, 1.37E-02) | 6.06E-01 |
| Free cholesterol in very large HDL | 2.61E-03 | (-8.34E-03, 1.35E-02) | 6.41E-01 |
| Triglycerides in very large HDL | -4.89E-03 | (-1.57E-02, 5.88E-03) | 3.74E-01 |
| Concentration of large HDL particles | 1.34E-02 | (2.30E-03, 2.45E-02) | 1.82E-02 |
| Total lipids in large HDL | 1.34E-02 | (2.27E-03, 2.44E-02) | 1.84E-02 |
| Phospholipids in large HDL | 1.44E-02 | (3.24E-03, 2.55E-02) | 1.15E-02 |
| Total cholesterol in large HDL | 1.23E-02 | (1.31E-03, 2.34E-02) | 2.85E-02 |
| Cholesterol esters in large HDL | 1.24E-02 | (1.32E-03, 2.34E-02) | 2.84E-02 |
| Free cholesterol in large HDL | 1.22E-02 | (1.20E-03, 2.32E-02) | 3.00E-02 |
| Triglycerides in large HDL | 8.39E-03 | (-2.80E-03, 1.96E-02) | 1.42E-01 |
| Concentration of medium HDL particles | 1.38E-02 | (2.72E-03, 2.49E-02) | 1.48E-02 |
| Total lipids in medium HDL | 1.42E-02 | (3.08E-03, 2.53E-02) | 1.24E-02 |
| Phospholipids in medium HDL | 1.35E-02 | (2.37E-03, 2.47E-02) | 1.77E-02 |
| Total cholesterol in medium HDL | 1.55E-02 | (4.49E-03, 2.65E-02) | 5.89E-03 |
| Cholesterol esters in medium HDL | 1.53E-02 | (4.36E-03, 2.63E-02) | 6.29E-03 |
| Free cholesterol in medium HDL | 1.58E-02 | (4.67E-03, 2.69E-02) | 5.49E-03 |
| Triglycerides in medium HDL | -5.33E-03 | (-1.61E-02, 5.48E-03) | 3.34E-01 |
| Concentration of small HDL particles | 1.42E-02 | (3.38E-03, 2.50E-02) | 1.02E-02 |
| Total lipids in small HDL | 1.50E-02 | (4.16E-03, 2.58E-02) | 6.75E-03 |
| Phospholipids in small HDL | 8.93E-03 | (-1.89E-03, 1.97E-02) | 1.06E-01 |
| Total cholesterol in small HDL | 1.91E-02 | (8.35E-03, 2.98E-02) | 5.11E-04 |
| Cholesterol esters in small HDL | 1.84E-02 | (7.68E-03, 2.91E-02) | 7.93E-04 |
| Free cholesterol in small HDL | 1.20E-02 | (1.20E-03, 2.28E-02) | 2.97E-02 |
| Triglycerides in small HDL | -8.96E-03 | (-1.97E-02, 1.76E-03) | 1.02E-01 |
| Phospholipids to total lipids ratio in very large HDL | 5.14E-03 | (-5.74E-03, 1.60E-02) | 3.55E-01 |
| Total cholesterol to total lipids ratio in very large HDL | -3.87E-03 | (-1.47E-02, 6.98E-03) | 4.85E-01 |
| Cholesterol esters to total lipids ratio in very large HDL | -2.93E-03 | (-1.37E-02, 7.83E-03) | 5.94E-01 |
| Free cholesterol to total lipids ratio in very large HDL | -2.30E-03 | (-1.32E-02, 8.58E-03) | 6.79E-01 |
| Triglycerides to total lipids ratio in very large HDL | -8.15E-03 | (-1.90E-02, 2.71E-03) | 1.42E-01 |
| Phospholipids to total lipids ratio in large HDL | -1.19E-02 | (-2.30E-02, -7.74E-04) | 3.63E-02 |
| Total cholesterol to total lipids ratio in large HDL | 1.34E-02 | (2.40E-03, 2.44E-02) | 1.71E-02 |
| Cholesterol esters to total lipids ratio in large HDL | 1.11E-02 | (1.12E-04, 2.21E-02) | 4.80E-02 |
| Free cholesterol to total lipids ratio in large HDL | 1.62E-02 | (5.31E-03, 2.72E-02) | 3.66E-03 |
| Triglycerides to total lipids ratio in large HDL | -3.20E-03 | (-1.42E-02, 7.80E-03) | 5.69E-01 |
| Phospholipids to total lipids ratio in medium HDL | -1.21E-02 | (-2.28E-02, -1.42E-03) | 2.65E-02 |
| Total cholesterol to total lipids ratio in medium HDL | 1.63E-02 | (5.63E-03, 2.70E-02) | 2.82E-03 |
| Cholesterol esters to total lipids ratio in medium HDL | 1.40E-02 | (3.34E-03, 2.47E-02) | 1.02E-02 |
| Free cholesterol to total lipids ratio in medium HDL | 1.80E-02 | (7.02E-03, 2.90E-02) | 1.37E-03 |
| Triglycerides to total lipids ratio in medium HDL | -1.72E-02 | (-2.79E-02, -6.50E-03) | 1.68E-03 |
| Phospholipids to total lipids ratio in small HDL | -7.31E-03 | (-1.81E-02, 3.43E-03) | 1.82E-01 |
| Total cholesterol to total lipids ratio in small HDL | 1.20E-02 | (1.30E-03, 2.27E-02) | 2.81E-02 |
| Cholesterol esters to total lipids ratio in small HDL | 1.10E-02 | (2.94E-04, 2.17E-02) | 4.43E-02 |
| Free cholesterol to total lipids ratio in small HDL | 4.78E-04 | (-1.03E-02, 1.12E-02) | 9.31E-01 |
| Triglycerides to total lipids ratio in small HDL | -1.49E-02 | (-2.56E-02, -4.23E-03) | 6.34E-03 |
| **Lipoprotein Particle** |  |  |  |
| Mean diameter for VLDL particles | -9.92E-03 | (-2.07E-02, 8.98E-04) | 7.26E-02 |
| Mean diameter for LDL particles | -6.10E-03 | (-1.70E-02, 4.84E-03) | 2.75E-01 |
| Mean diameter for HDL particles | 9.16E-03 | (-1.95E-03, 2.03E-02) | 1.06E-01 |
| Serum total cholesterol | 3.17E-03 | (-7.83E-03, 1.42E-02) | 5.72E-01 |
| Total cholesterol in VLDL | -1.43E-02 | (-2.50E-02, -3.58E-03) | 9.08E-03 |
| Remnant cholesterol (non-HDL, non-LDL -cholesterol) | -1.08E-02 | (-2.16E-02, 8.37E-05) | 5.21E-02 |
| Total cholesterol in LDL | 2.45E-03 | (-8.36E-03, 1.33E-02) | 6.57E-01 |
| Total cholesterol in HDL | 1.58E-02 | (4.81E-03, 2.69E-02) | 4.97E-03 |
| Total cholesterol in HDL2 | 1.59E-02 | (4.87E-03, 2.69E-02) | 4.78E-03 |
| Total cholesterol in HDL3 | 7.92E-03 | (-3.03E-03, 1.89E-02) | 1.57E-01 |
| Esterified cholesterol | 5.28E-03 | (-5.72E-03, 1.63E-02) | 3.47E-01 |
| Free cholesterol | -1.90E-03 | (-1.29E-02, 9.11E-03) | 7.36E-01 |
| Serum total triglycerides | -1.35E-02 | (-2.42E-02, -2.80E-03) | 1.36E-02 |
| Triglycerides in VLDL | -1.35E-02 | (-2.43E-02, -2.80E-03) | 1.36E-02 |
| Triglycerides in LDL | -6.76E-03 | (-1.77E-02, 4.22E-03) | 2.28E-01 |
| Triglycerides in HDL | -6.43E-03 | (-1.73E-02, 4.39E-03) | 2.44E-01 |
| Total phosphoglycerides | 5.15E-03 | (-5.91E-03, 1.62E-02) | 3.62E-01 |
| Ratio of triglycerides to phosphoglycerides | -1.58E-02 | (-2.66E-02, -5.07E-03) | 4.02E-03 |
| Phosphatidylcholine and other cholines | 8.12E-03 | (-3.16E-03, 1.94E-02) | 1.58E-01 |
| Sphingomyelins | 1.71E-03 | (-9.69E-03, 1.31E-02) | 7.68E-01 |
| Total cholines | 9.55E-03 | (-1.73E-03, 2.08E-02) | 9.73E-02 |
| **Apolipoproteins** |  |  |  |
| Apolipoprotein A-I | 1.27E-02 | (1.67E-03, 2.38E-02) | 2.43E-02 |
| Apolipoprotein B | -1.02E-02 | (-2.10E-02, 5.58E-04) | 6.34E-02 |
| Ratio of apolipoprotein B to apolipoprotein A-I | -1.69E-02 | (-2.76E-02, -6.13E-03) | 2.14E-03 |
| **Fatty Acids** |  |  |  |
| Total fatty acids | -5.71E-03 | (-1.66E-02, 5.18E-03) | 3.04E-01 |
| Estimated degree of unsaturation | 1.45E-02 | (3.78E-03, 2.52E-02) | 8.10E-03 |
| 22:6, docosahexaenoic acid | 5.18E-03 | (-5.83E-03, 1.62E-02) | 3.56E-01 |
| 18:2, linoleic acid | 3.09E-03 | (-7.73E-03, 1.39E-02) | 5.75E-01 |
| Omega-3 fatty acids | 5.08E-03 | (-5.92E-03, 1.61E-02) | 3.65E-01 |
| Omega-6 fatty acids | 3.89E-03 | (-7.03E-03, 1.48E-02) | 4.85E-01 |
| Polyunsaturated fatty acids | 4.46E-03 | (-6.51E-03, 1.54E-02) | 4.26E-01 |
| Monounsaturated fatty acids; 16:1, 18:1 | -1.19E-02 | (-2.27E-02, -1.11E-03) | 3.09E-02 |
| Saturated fatty acids | -5.37E-03 | (-1.62E-02, 5.48E-03) | 3.32E-01 |
| Ratio of 22:6 docosahexaenoic acid to total fatty acids | 1.06E-02 | (-1.68E-04, 2.14E-02) | 5.40E-02 |
| Ratio of 18:2 linoleic acid to total fatty acids | 1.22E-02 | (1.51E-03, 2.30E-02) | 2.55E-02 |
| Ratio of omega-3 fatty acids to total fatty acids | 1.11E-02 | (3.92E-04, 2.18E-02) | 4.24E-02 |
| Ratio of omega-6 fatty acids to total fatty acids | 1.42E-02 | (3.46E-03, 2.49E-02) | 9.63E-03 |
| Ratio of polyunsaturated fatty acids to total fatty acids | 1.53E-02 | (4.60E-03, 2.60E-02) | 5.15E-03 |
| Ratio of monounsaturated fatty acids to total fatty acids | -1.69E-02 | (-2.77E-02, -6.20E-03) | 2.03E-03 |
| Ratio of saturated fatty acids to total fatty acids | -1.09E-03 | (-1.18E-02, 9.62E-03) | 8.42E-01 |
| **Glycolysis Related Metabolites** |  |  |  |
| Glucose | -7.18E-03 | (-1.79E-02, 3.51E-03) | 1.88E-01 |
| Lactate | -2.54E-02 | (-3.60E-02, -1.48E-02) | 2.95E-06 |
| Pyruvate | -1.65E-02 | (-2.74E-02, -5.66E-03) | 2.92E-03 |
| Citrate | -3.82E-03 | (-1.49E-02, 7.29E-03) | 5.00E-01 |
| Glycerol | -2.47E-02 | (-3.63E-02, -1.32E-02) | 2.88E-05 |
| **Amino Acids** |  |  |  |
| Alanine | -6.08E-03 | (-1.68E-02, 4.61E-03) | 2.65E-01 |
| Glutamine | 6.99E-04 | (-1.01E-02, 1.14E-02) | 8.99E-01 |
| Glycine | -6.45E-03 | (-1.77E-02, 4.79E-03) | 2.61E-01 |
| Histidine | 1.83E-02 | (7.42E-03, 2.92E-02) | 1.01E-03 |
| Isoleucine | -1.66E-02 | (-2.75E-02, -5.63E-03) | 3.04E-03 |
| Leucine | -2.09E-03 | (-1.33E-02, 9.14E-03) | 7.15E-01 |
| Valine | 5.80E-03 | (-5.16E-03, 1.68E-02) | 3.00E-01 |
| Phenylalanine | -8.62E-03 | (-1.93E-02, 2.08E-03) | 1.15E-01 |
| Tyrosine | 6.23E-03 | (-4.46E-03, 1.69E-02) | 2.53E-01 |
| **Ketone Bodies** |  |  |  |
| Acetate | 4.17E-03 | (-6.61E-03, 1.50E-02) | 4.48E-01 |
| Acetoacetate | -2.35E-03 | (-1.32E-02, 8.51E-03) | 6.71E-01 |
| 3-hydroxybutyrate | -2.42E-03 | (-1.32E-02, 8.40E-03) | 6.62E-01 |
| **Fluid Balance** |  |  |  |
| Creatinine | -2.26E-02 | (-3.37E-02, -1.15E-02) | 6.91E-05 |
| Albumin | 4.35E-03 | (-6.47E-03, 1.52E-02) | 4.31E-01 |
| **Inflammation** |  |  |  |
| Glycoprotein acetyls | -2.34E-02 | (-3.40E-02, -1.27E-02) | 1.76E-05 |

# Table S4. Association between key metabolites and baseline ABI in univariate analysis.

| **Metabolites** | **Model 1**  **(adjusted for age and gender)** | | |  | **Model 2**  **(Model 1+SBP+Smoking+HDL-cholesterol +Total cholesterol+BMI+HbA1c)** | | |  | **Model 3a (Model2+eGFR+diabetes duration+lowering-lipid drug use)** | | |
| --- | --- | --- | --- | --- | --- | --- | --- | --- | --- | --- | --- |
|  | Beta | 95% CI | *p* value |  | Beta | 95% CI | *p* value |  | Beta | 95% CI | *p* value |
| Lactate * | -0.025 | (-0.036, -0.015) | 2.95E-06 |  | -0.019 | (-0.029, -0.008) | 6.33E-04 |  | -0.020 | (0.031, -0.010) | 2.11E-04 |
| Glycerol * | -0.025 | (-0.036, -0.013) | 2.88E-05 |  | -0.018 | (-0.031, -0.006) | 3.44E-03 |  | -0.018 | (0.031, 0.006) | 4.55E-03 |
| Creatinine * | -0.023 | (-0.034, -0.012) | 6.91E-05 |  | -0.017 | (-0.029, -0.006) | 2.70E-03 |  | -0.015 | (0.031, 0.000) | 5.42E-02 |
| Glycoprotein acetyls * | -0.023 | (-0.034, -0.013) | 1.76E-05 |  | -0.014 | (-0.026, -0.001) | 2.80E-02 |  | -0.011 | (0.024, 0.001) | 6.91E-02 |

*: metabolites associated with baseline ABI which were also reported in LASSO analysis

# Table S5. Association between key metabolites and baseline ABI as estimated by LASSO.

| **Model 1**  **(adjusted for age and gender)** |  |  | **Model 2**  **(Model 1+SBP+Smoking+HDL-cholesterol+Total cholesterol+BMI+HbA1c)** |  |
| --- | --- | --- | --- | --- |
| Metabolites | *Beta* |  | Metabolites | *Beta* |
| Total lipids in small HDL | 0.006 |  | Free cholesterol to total lipids ratio in medium LDL | -0.004 |
| Total cholesterol in small HDL | 0.004 |  | Concentration of small HDL particles | 0.003 |
| Lactate * | -0.010 |  | Total cholesterol in small HDL | 0.006 |
| Glycerol * | -0.007 |  | Mean diameter for VLDL particles | -0.002 |
| Histidine | 0.008 |  | Lactate | -0.011 |
| Creatinine * | -0.008 |  | Glycerol | -0.002 |
| Glycoprotein acetyls * | -0.006 |  | Histidine | 0.009 |
|  |  |  | Creatinine | -0.009 |

*: metabolites associated with baseline ABI which were also reported in univariate analysis

# Table S6. Association between the four key metabolites and follow-up ABI in univariate analysis.

| **Metabolites** |  | **ABI_Y10 (adjusted for baseline ABI)** |  |  |  | **ABI_Y4 (adjusted for baseline ABI)** |  |  |
| --- | --- | --- | --- | --- | --- | --- | --- | --- |
|  |  | Beta | 95%CI | *p* value |  | Beta | 95%CI | *p* value |
| Lactate | Model 1 | -0.020 | (-0.036, -0.005) | 1.21E-02 |  | -0.014 | (-0.024, -0.003) | 9.99E-03 |
|  | Model 2 | -0.022 | (-0.039, -0.006) | 7.59E-03 |  | -0.013 | (-0.023, -0.002) | 2.27E-02 |
| Glycerol | Model 1 | -0.007 | (-0.025, 0.010) | 3.95E-01 |  | -0.008 | (-0.020, 0.003) | 1.41E-01 |
|  | Model 2 | -0.009 | (-0.028, 0.010) | 3.43E-01 |  | -0.009 | (-0.021, 0.003) | 1.64E-01 |
| Creatinine | Model 1 | 0.005 | (-0.011, 0.022) | 5.47E-01 |  | -0.010 | (-0.021, 0.001) | 6.28E-02 |
|  | Model 2 | 0.007 | (-0.010, 0.024) | 4.29E-01 |  | -0.010 | (-0.021, 0.002) | 1.02E-01 |
| Glycoprotein acetyls | Model 1 | -0.016 | (-0.031, 0.000) | 5.13E-02 |  | -0.009 | (-0.019, 0.002) | 9.90E-02 |
|  | Model 2 | -0.013 | (-0.031, 0.006) | 1.72E-01 |  | -0.003 | (-0.015, 0.010) | 6.67E-01 |

Model 1: adjusted for age and gender, and Model 2: adjusted for age, gender, SBP, smoking, HDL-cholesterol, total cholesterol, BMI and HbA1c

# Table S7. Association between key metabolites and prevalent CVD at baseline in univariate analysis.

| **Metabolites** | **Model 1**  **(adjusted for age and gender)** | | |  | **Model 2**  **(Model 1+SBP+Smoking+HDL-** **cholesterol+Total cholesterol+BMI+HbA1c)** | | |  | **Model 3a**  **(Model2+eGFR+diabetes duration+lowering-lipid drug use)** | | |
| --- | --- | --- | --- | --- | --- | --- | --- | --- | --- | --- | --- |
|  | OR | 95% CI | *p* value |  | OR | 95% CI | *p* value |  | OR | 95% CI | *p* value |
| Phospholipids to total lipids ratio in small VLDL | 0.75 | (0.65, 0.86) | 7.29E-05 |  | 0.91 | (0.75, 1.10) | 3.47E-01 |  | 0.92 | (0.76, 1.12) | 3.95E-01 |
| Concentration of large HDL particles | 0.71 | (0.61, 0.82) | 6.32E-06 |  | 1.10 | (0.81, 1.50) | 5.34E-01 |  | 1.22 | (0.89, 1.68) | 2.29E-01 |
| Total lipids in large HDL | 0.71 | (0.61, 0.82) | 6.79E-06 |  | 1.11 | (0.82, 1.51) | 5.12E-01 |  | 1.22 | (0.89, 1.69) | 2.19E-01 |
| Phospholipids in large HDL | 0.70 | (0.60, 0.81) | 2.26E-06 |  | 1.04 | (0.78, 1.40) | 7.94E-01 |  | 1.14 | (0.84, 1.55) | 4.02E-01 |
| Total cholesterol in large HDL | 0.72 | (0.61, 0.83) | 1.62E-05 |  | 1.14 | (0.84, 1.56) | 3.98E-01 |  | 1.25 | (0.91, 1.73) | 1.69E-01 |
| Cholesterol esters in large HDL | 0.71 | (0.61, 0.83) | 1.41E-05 |  | 1.13 | (0.83, 1.55) | 4.30E-01 |  | 1.25 | (0.91, 1.73) | 1.75E-01 |
| Free cholesterol in large HDL | 0.72 | (0.62, 0.84) | 2.88E-05 |  | 1.16 | (0.87, 1.56) | 3.18E-01 |  | 1.24 | (0.92, 1.69) | 1.61E-01 |
| Concentration of medium HDL particles * | 0.58 | (0.50, 0.68) | 4.05E-12 |  | 0.61 | (0.49, 0.74) | 2.34E-06 |  | 0.60 | (0.48, 0.75) | 5.37E-06 |
| Total lipids in medium HDL | 0.58 | (0.50, 0.68) | 3.54E-12 |  | 0.60 | (0.49, 0.74) | 2.60E-06 |  | 0.60 | (0.48, 0.75) | 6.00E-06 |
| Phospholipids in medium HDL | 0.59 | (0.50, 0.68) | 8.19E-12 |  | 0.61 | (0.50, 0.75) | 3.26E-06 |  | 0.61 | (0.49, 0.75) | 6.86E-06 |
| Total cholesterol in medium HDL | 0.59 | (0.50, 0.68) | 3.36E-12 |  | 0.59 | (0.47, 0.74) | 6.51E-06 |  | 0.59 | (0.46, 0.75) | 1.61E-05 |
| Cholesterol esters in medium HDL | 0.59 | (0.50, 0.68) | 3.11E-12 |  | 0.59 | (0.47, 0.74) | 5.74E-06 |  | 0.59 | (0.46, 0.75) | 1.61E-05 |
| Free cholesterol in medium HDL | 0.59 | (0.51, 0.69) | 1.26E-11 |  | 0.62 | (0.50, 0.78) | 2.62E-05 |  | 0.62 | (0.49, 0.78) | 3.94E-05 |
| Concentration of small HDL particles | 0.73 | (0.63, 0.83) | 7.28E-06 |  | 0.78 | (0.67, 0.90) | 1.07E-03 |  | 0.75 | (0.64, 0.88) | 3.52E-04 |
| Total lipids in small HDL | 0.72 | (0.63, 0.83) | 4.95E-06 |  | 0.78 | (0.67, 0.91) | 1.50E-03 |  | 0.76 | (0.64, 0.88) | 5.15E-04 |
| Phospholipids in small HDL * | 0.68 | (0.59, 0.78) | 1.13E-07 |  | 0.71 | (0.61, 0.83) | 1.30E-05 |  | 0.69 | (0.58, 0.81) | 5.23E-06 |
| Free cholesterol in small HDL | 0.71 | (0.62, 0.82) | 1.94E-06 |  | 0.78 | (0.66, 0.90) | 1.25E-03 |  | 0.74 | (0.63, 0.87) | 2.84E-04 |
| Phospholipids to total lipids ratio in medium HDL | 1.41 | (1.23, 1.62) | 6.76E-07 |  | 1.18 | (1.00, 1.40) | 4.55E-02 |  | 1.16 | (0.96, 1.38) | 1.05E-01 |
| Total cholesterol to total lipids ratio in medium HDL | 0.71 | (0.62, 0.81) | 4.42E-07 |  | 0.87 | (0.71, 1.08) | 2.05E-01 |  | 0.89 | (0.72, 1.11) | 3.04E-01 |
| Cholesterol esters to total lipids ratio in medium HDL | 0.74 | (0.65, 0.84) | 8.92E-06 |  | 0.92 | (0.76, 1.12) | 3.75E-01 |  | 0.94 | (0.77, 1.16) | 5.66E-01 |
| Free cholesterol to total lipids ratio in medium HDL | 0.70 | (0.61, 0.80) | 3.23E-07 |  | 0.87 | (0.72, 1.04) | 1.32E-01 |  | 0.85 | (0.70, 1.04) | 1.11E-01 |
| Triglycerides to total lipids ratio in medium HDL | 1.33 | (1.16, 1.52) | 2.84E-05 |  | 1.00 | (0.81, 1.24) | 9.81E-01 |  | 1.01 | (0.81, 1.25) | 9.49E-01 |
| Triglycerides to total lipids ratio in small HDL | 1.41 | (1.23, 1.62) | 6.48E-07 |  | 1.15 | (0.94, 1.40) | 1.77E-01 |  | 1.09 | (0.88, 1.34) | 4.21E-01 |
| Mean diameter for HDL particles | 0.73 | (0.63, 0.85) | 3.47E-05 |  | 1.10 | (0.85, 1.42) | 4.90E-01 |  | 1.22 | (0.93, 1.60) | 1.46E-01 |
| Total cholesterol in HDL | 0.65 | (0.55, 0.75) | 2.02E-08 |  | 0.75 | (0.52, 1.09) | 1.29E-01 |  | 0.84 | (0.57, 1.24) | 3.77E-01 |
| Total cholesterol in HDL2 | 0.64 | (0.54, 0.74) | 6.23E-09 |  | 0.69 | (0.48, 0.99) | 4.22E-02 |  | 0.78 | (0.53, 1.14) | 1.96E-01 |
| Total phosphoglycerides | 0.74 | (0.64, 0.86) | 4.50E-05 |  | 0.72 | (0.58, 0.90) | 3.55E-03 |  | 0.69 | (0.55, 0.87) | 1.62E-03 |
| Ratio of triglycerides to phosphoglycerides | 1.32 | (1.15, 1.50) | 5.37E-05 |  | 1.01 | (0.85, 1.21) | 8.94E-01 |  | 0.96 | (0.80, 1.15) | 6.84E-01 |
| Phosphatidylcholine and other cholines | 0.71 | (0.61, 0.82) | 4.91E-06 |  | 0.69 | (0.54, 0.88) | 2.56E-03 |  | 0.66 | (0.51, 0.85) | 1.49E-03 |
| Total cholines | 0.70 | (0.61, 0.81) | 2.66E-06 |  | 0.65 | (0.49, 0.85) | 1.91E-03 |  | 0.65 | (0.48, 0.86) | 2.63E-03 |
| Apolipoprotein A-I | 0.66 | (0.56, 0.76) | 4.82E-08 |  | 0.65 | (0.46, 0.91) | 1.33E-02 |  | 0.67 | (0.47, 0.96) | 3.12E-02 |
| Ratio of apolipoprotein B to apolipoprotein A-I | 1.33 | (1.17, 1.52) | 2.62E-05 |  | 1.06 | (0.75, 1.49) | 7.42E-01 |  | 0.91 | (0.63, 1.31) | 6.13E-01 |
| Lactate * | 1.29 | (1.13, 1.47) | 1.27E-04 |  | 1.18 | (1.03, 1.35) | 2.06E-02 |  | 1.20 | (1.04, 1.38) | 1.24E-02 |
| Glycerol * | 1.34 | (1.16, 1.54) | 7.32E-05 |  | 1.21 | (1.03, 1.42) | 2.18E-02 |  | 1.16 | (0.97, 1.37) | 9.49E-02 |
| Creatinine * | 1.49 | (1.29, 1.74) | 2.24E-07 |  | 1.35 | (1.16, 1.59) | 1.40E-04 |  | 1.21 | (0.98, 1.51) | 8.38E-02 |
| Glycoprotein acetyls * | 1.31 | (1.15, 1.50) | 7.81E-05 |  | 1.12 | (0.96, 1.31) | 1.57E-01 |  | 1.04 | (0.89, 1.23) | 6.07E-01 |

*: metabolites associated with prevalent CVD which were also reported in LASSO analysis.


# Table S8. Association between key metabolites and prevalent CVD at baseline as estimated by LASSO.

| **Model 1**  **(adjusted for age and gender)** | |  | **Model 2**  **(Model 1+SBP+Smoking+HDL-cholesterol +Total cholesterol+BMI+HbA1c)** |  |
| --- | --- | --- | --- | --- |
| Metabolites | OR |  | Metabolites | OR |
| Free cholesterol to total lipids ratio in chylomicrons and extremely large VLDL | 0.97 |  | Free cholesterol to total lipids ratio in chylomicrons and extremely large VLDL | 0.98 |
| Cholesterol esters to total lipids ratio in IDL | 0.93 |  | Phospholipids in small HDL | 0.78 |
| Concentration of medium HDL particles * | 0.84 |  | Phospholipids to total lipids ratio in large HDL | 0.99 |
| Triglycerides in medium HDL | 0.98 |  | Triglycerides to total lipids ratio in large HDL | 1.01 |
| Phospholipids in small HDL * | 0.87 |  | Phospholipids to total lipids ratio in small HDL | 0.84 |
| Triglycerides to total lipids ratio in large HDL | 1.01 |  | Total cholesterol in HDL3 | 1.01 |
| Phospholipids to total lipids ratio in small HDL | 0.88 |  | Ratio of 22:6 docosahexaenoic acid to total fatty acids | 0.99 |
| 22:6, docosahexaenoic acid | 0.98 |  | Lactate | 1.01 |
| Omega-3 fatty acids | 0.95 |  | Pyruvate | 1.03 |
| Lactate * | 1.01 |  | Glycerol | 1.09 |
| Pyruvate | 1.08 |  | Alanine | 1.10 |
| Glycerol * | 1.12 |  | Glycine | 0.91 |
| Alanine | 1.07 |  | Histidine | 0.94 |
| Glycine | 0.91 |  | Leucine | 1.00 |
| Histidine | 0.92 |  | Phenylalanine | 1.05 |
| Leucine | 1.00 |  | Tyrosine | 1.02 |
| Phenylalanine | 1.08 |  | Acetate | 0.84 |
| Tyrosine | 1.03 |  | Acetoacetate | 0.95 |
| Acetate | 0.84 |  | Creatinine | 1.21 |
| Acetoacetate | 0.93 |  | Albumin | 0.96 |
| Creatinine * | 1.21 |  | Glycoprotein acetyls | 1.10 |
| Albumin | 0.95 |  |  |  |
| Glycoprotein acetyls * | 1.10 |  |  |  |

*: metabolites associated with prevalent CVD which were also reported in univariate analysis

# Table S9. Association between the four key metabolites and incident CVD over 10 years in univariate analysis.

| **Metabolites** |  | **Overall CVD in 10 years (including newly incident events and recurrent events)** |  |  |  | **Newly incident CVD in 10 years** |  |  |
| --- | --- | --- | --- | --- | --- | --- | --- | --- |
|  |  | OR | 95%CI | *p* value |  | OR | 95%CI | *p* value |
| Lactate | Model 1 | 1.26 | (1.10, 1.44) | 9.35E-04 |  | 1.29 | (1.07, 1.55) | 7.23E-03 |
|  | Model 2 | 1.17 | (1.02, 1.35) | 2.62E-02 |  | 1.22 | (1.00, 1.48) | 4.19E-02 |
| Glycerol | Model 1 | 1.19 | (1.02, 1.39) | 2.30E-02 |  | 1.07 | (0.86, 1.33) | 5.32E-01 |
|  | Model 2 | 1.12 | (0.95, 1.31) | 1.67E-01 |  | 1.01 | (0.81, 1.27) | 9.00E-01 |
| Creatinine | Model 1 | 1.35 | (1.17, 1.56) | 5.89E-05 |  | 1.18 | (0.96, 1.43) | 1.03E-01 |
|  | Model 2 | 1.28 | (1.11, 1.49) | 1.13E-03 |  | 1.15 | (0.94, 1.40) | 1.67E-01 |
| Glycoprotein acetyls | Model 1 | 1.30 | (1.13, 1.49) | 2.29E-04 |  | 1.23 | (1.02, 1.49) | 2.67E-02 |
|  | Model 2 | 1.07 | (0.91, 1.25) | 4.31E-01 |  | 1.09 | (0.88, 1.36) | 4.18E-01 |

**Table S10. Association between the four baseline ABI-associated metabolites and overall incident CVD with adjustment for age, gender and prevalent CVD.**

| **Metabolites** | **ORs (95%CI)** | ***p* values** |
| --- | --- | --- |
| Lactate | 1.21 (1.05, 1.39) | 0.009 |
| Glycerol | 1.13 (0.97, 1.32) | 0.120 |
| Creatinine | 1.26 (1.09, 1.47) | 0.002 |
| Glycoprotein Acetyls | 1.24 (1.08, 1.43) | 0.002 |
